# Supplementary material for: Do sex differences in the prevalence of ECG abnormalities vary across ethnic groups living in the Netherlands? A cross-sectional analysis of the population-based HELIUS study
Source: BMJ Open. 2020 Sep 3;10(9):e039091. doi: 10.1136/bmjopen-2020-039091 (PMC7473628; doi:10.1136/bmjopen-2020-039091)
Supplement: Supplementary data [file bmjopen-2020-039091supp002.pdf]

Appendix Table 1. Classification of major and minor ECG abnormalities

**Major ECG abnormalities**
Atrial fibrillation or flutter
Ventricular rhythm
Sinus bradycardia + idioventricular rhythm
2nd degree atrioventricular conduction disturbances
3rd degree atrioventricular conduction disturbances
Ventricular preexcitation
Left bundle branch block (LBBB)
Right bundle branch block (RBBB)
Nonspecific ventricular conduction disturbances (QRS duration ≥120ms)
Severely prolonged QTc (Bazett) interval (men: QTc >470ms, women: QTc ≥480ms)
Severely shortened QTc (Bazett) interval (men: QTc <330ms, women: QTc <340ms)
Extreme axis deviation
Pathological Q waves
Microvoltages
T-wave abnormalities
ECG suggestive of cardiomyopathy
Miscellaneous (including ECG suggestive of Brugada syndrome)
**Minor ECG abnormalities**
Sinus tachycardia
Sinus bradycardia
Atrial rhythm
Frequent premature atrial contractions (PACs) and/or premature ventricular contractions (PVCs)
Atrial abnormalities
1st degree atrioventricular conduction disturbances
Left anterior fascicular block (LAFB)
Mild (110≤QRS<120ms) ventricular conduction disturbances, including incomplete LBBB and incomplete RBBB

Mildly prolonged QTc (Bazett) interval (men: QTc >450ms, women: QTc ≥460ms)
Mildly shortened QTc (Bazett) interval (men: QTc <360ms, women: QTc <370ms)
Left axis deviation
Right axis deviation
Indeterminate heart axis
Possible pathological Q waves
Left ventricular hypertrophy (LVH)

ECG, electrocardiogram.

Appendix Table 2. Baseline characteristics of 19,548 participants with ECG measurements, by sex and ethnicity

	Dutch		South-Asian Surinamese		African Surinamese	
	**Men (n=1,873)**	**Women (n=2,293)**	**Men (n=1,125)**	**Women (n=1,464)**	**Men (n=1,411)**	**Women (n=2,266)**
Age (years)	45.8 (13.6)	44.8 (14.0)	42.6 (13.2)	45.0 (13.1)	47.2 (12.9)	47.1 (12.3)
Family history of CVD (missing: n=217*)	418 (22.4)	536 (23.5)	399 (35.8)	583 (40.5)	231 (16.5)	473 (21.2)
Smoker						
Current	477 (25.5)	532 (23.2)	432 (38.4)	277 (18.9)	597 (42.3)	548 (24.2)
Past	722 (38.5)	842 (36.7)	177 (15.7)	159 (10.9)	291 (20.6)	400 (17.7)
Never	674 (36.0)	919 (40.1)	516 (45.9)	1,028 (70.2)	523 (37.1)	1,318 (58.2)
Pack-years of smoking (missing: n=191*)	3.4 (10.6)	2.9 (9.7)	8.9 (26.9)	1.7 (5.9)	8.3 (20.3)	2.5 (9.8)
Achieving physical activity norm (missing: n=27*)	1,365 (72.9)	1,792 (78.2)	645 (57.5)	729 (49.9)	983 (69.8)	1,270 (56.1)
Alcohol consumption (missing: n=115*)						
None or low	721 (38.6)	774 (33.9)	880 (78.6)	1,261 (86.7)	1,056 (75.6)	1,883 (83.7)
Moderate	884 (47.3)	925 (40.5)	151 (13.5)	149 (10.2)	260 (18.6)	278 (12.4)

High	264 (14.1)	587 (25.7)	89 (7.9)	44 (3.0)	80 (5.7)	89 (4.0)
BMI (kg/m^2; missing: n=15*)	25.0 (3.7)	24.2 (4.3)	25.7 (4.1)	26.4 (5.2)	26.2 (4.3)	28.6 (5.9)
CKD risk (missing: n=63*)						
Low	1,804 (96.8)	2,216 (96.9)	1,046 (93.2)	1,365 (93.5)	1,344 (95.5)	2,139 (94.6)
Moderate	49 (2.6)	64 (2.8)	62 (5.5)	78 (5.3)	53 (3.8)	100 (4.4)
High	10 (0.5)	7 (0.3)	14 (1.2)	17 (1.2)	11 (0.8)	21 (0.9)
Hypertension	618 (33.0)	471 (20.5)	429 (38.1)	527 (36.0)	660 (46.8)	1,072 (47.3)
Hypercholesterolemia	1,314 (70.2)	1,374 (59.9)	934 (83.0)	1,120 (76.5)	889 (63.0)	1,447 (63.9)
Diabetes	76 (4.1)	40 (1.7)	190 (16.9)	209 (14.3)	149 (10.6)	237 (10.5)
Use of psychotropic medication (missing: n=4*)	88 (4.7)	190 (8.3)	57 (5.1)	100 (6.8)	53 (3.8)	105 (4.6)

	**Ghanaian**		**Turkish**		**Moroccan**	
	**Men (n=822)**	**Women (n=1,321)**	**Men (n=1,451)**	**Women (n=1,769)**	**Men (n=1,407)**	**Women (n=2,256)**
Age (years)	46.3 (11.6)	43.0 (10.7)	39.8 (11.9)	39.1 (12.0)	41.6 (12.7)	39.1 (12.8)
Family history of CVD (missing: n=217*)	36 (4.4)	53 (4.1)	382 (26.7)	615 (35.2)	171 (12.3)	351 (15.8)
Smoker						
Current	60 (7.3)	32 (2.4)	602 (41.5)	521 (29.5)	371 (26.4)	122 (5.4)

Past	102 (12.4)	66 (5.0)	354 (24.4)	207 (11.7)	375 (26.7)	79 (3.5)
Never	660 (80.3)	1,223 (92.6)	495 (34.1)	1,041 (58.8)	661 (47.0)	2,055 (91.1)
Pack-years of smoking (missing: n=191*)	0.6 (4.2)	0.2 (2.3)	6.9 (14.8)	2.9 (7.2)	3.8 (9.9)	0.3 (1.9)
Achieving physical activity norm (missing: n=27*)	516 (62.8)	626 (47.4)	720 (49.8)	618 (35.0)	791 (56.4)	928 (41.2)
Alcohol consumption (missing: n=115*)						
None or low	711 (87.6)	1,184 (90.5)	1,291 (89.8)	1,679 (95.3)	1,322 (94.4)	2,204 (98.0)
Moderate	98 (12.1)	103 (7.9)	87 (6.1)	62 (3.5)	46 (3.3)	32 (1.4)
High	3 (0.4)	22 (1.7)	59 (4.1)	20 (1.1)	33 (2.4)	12 (0.5)
BMI (kg/m^2; missing: n=15*)	26.6 (3.6)	29.5 (5.3)	27.7 (4.4)	28.9 (6.5)	26.7 (4.0)	28.0 (5.7)
CKD risk (missing: n=63*)						
Low	770 (94.2)	1,213 (92.2)	1,379 (95.4)	1,645 (93.3)	1,341 (95.7)	2,111 (93.7)
Moderate	37 (4.5)	87 (6.6)	57 (3.9)	106 (6.0)	46 (3.3)	120 (5.3)
High	10 (1.2)	16 (1.2)	9 (0.6)	12 (0.7)	14 (1.0)	22 (1.0)
Hypertension	494 (60.1)	673 (50.9)	427 (29.4)	407 (23.0)	398 (28.3)	444 (19.7)
Hypercholesterolemia	551 (67.0)	792 (60.0)	1,120 (77.2)	1,130 (63.9)	944 (67.1)	1,284 (56.9)

Diabetes	115 (14.0)	110 (8.3)	138 (9.5)	134 (7.6)	161 (11.4)	222 (9.8)
Use of psychotropic medication	24 (2.9)	41 (3.1)	88 (6.1)	139 (7.9)	87 (6.2)	104 (4.6)
(missing: n=4*)						

Data are presented as means (standard deviations) or frequencies (percentages).

BMI, body mass index; CKD, chronic kidney disease; CVD, cardiovascular disease.

* From total study population (n=19,458).

Appendix Table 3. Number of cases and age-adjusted prevalence of any minor ECG abnormality by sex in the total population and by ethnic group, and the odds of minor ECG abnormalities in women compared to men, overall and with an interaction term for sex and ethnicity

	**Men**	**Women**	**Model 1**				**Model 2**			
	**(n of cases, %[a])**	**(n of cases, %[a])**	**OR (95% CI)**	***p*-value**	**Ratio of ORs (95% CI)***	***p*-value**	**OR (95% CI)**	***p*-value**	**Ratio of ORs (95% CI)***	***p*-value**
Overall	3,227 (39.8)	2,684 (23.8)	0.45 (0.42-0.48)[b]	*<0.001*	NA	NA	0.46 (0.43-0.49)[b]	*<0.001*	NA	NA
Dutch	829 (44.3)	636 (27.6)	0.49 (0.43-0.55)	*<0.001*	Reference	NA	0.50 (0.44-0.57)	*<0.001*	Reference	NA
SA Surinamese	316 (28.5)	269 (17.9)	0.56 (0.46-0.67)	*<0.001*	1.15 (0.92-1.44)	0.22	0.57 (0.47-0.68)	*<0.001*	1.13 (0.90-1.42)	0.28
African Surinamese	647 (45.5)	638 (28.4)	0.46 (0.40-0.53)	*<0.001*	0.95 (0.78-1.15)	0.59	0.46 (0.40-0.53)	*<0.001*	0.93 (0.76-1.12)	0.43
Ghanaian	455 (55.7)	452 (35.6)	0.43 (0.36-0.52)	*<0.001*	0.89 (0.72-1.11)	0.31	0.44 (0.36-0.52)	*<0.001*	0.87 (0.70-1.09)	0.22
Turkish	468 (32.4)	283 (16.2)	0.40 (0.34-0.48)	*<0.001*	0.83 (0.67-1.02)	0.08	0.40 (0.34-0.47)	*<0.001*	0.80 (0.64-0.99)	*0.04*
Moroccan	512 (36.5)	406 (18.3)	0.39 (0.34-0.46)	*<0.001*	0.81 (0.66-0.99)	*0.04*	0.39 (0.34-0.46)	*<0.001*	0.78 (0.64-0.96)	*0.02*

CI, confidence interval; NA, not applicable; OR, odds ratio; SA, South-Asian.

Significant *p*-values ($p<0.05$) are printed in italic.

Model 1: adjusted for age.

Model 2: adjusted for age, hypertension, hypercholesterolemia, diabetes, and smoking status.

[a] Age-adjusted prevalence.

[b] Additionally adjusted for ethnicity.

* Measure of effect modification on multiplicative scale (statistical interaction term).

Appendix Table 4a. Age-adjusted prevalence of a selection of common ECG abnormalities (for majors: prevalence ≥1% in total study population; for minors: top 5 of abnormalities with highest prevalence in total study population), shown by major and minor ECG abnormality categories, by sex and ethnicity, ordered by highest to lowest overall prevalence

	**Major ECG abnormalities**		**Minor ECG abnormalities**				
	**T-wave abnormalities**	**ECG suggestive of cardiomyopathy**	**LVH**	**Sinus bradycardia**	**1st degree atrioventricular conduction disturbances**	**Mild ventricular conduction disturbances[a]**	**Mildly prolonged QTc (Bazett) interval[b]**
All							
Men	133 (1.6)	90 (1.1)	1,088 (13.5)	723 (9.0)	532 (6.5)	609 (7.5)	173 (2.1)
Women	137 (1.2)	124 (1.1)	986 (8.8)	473 (4.2)	344 (3.1)	133 (1.2)	393 (3.5)
Dutch							
Men	7 (0.4)	10 (0.5)	181 (9.8)	269 (14.3)	121 (6.3)	254 (13.6)	57 (3.0)
Women	12 (0.5)	15 (0.6)	114 (5.0)	218 (9.4)	72 (3.1)	55 (2.4)	69 (3.1)
South-Asian Surinamese							
Men	17 (1.9)	13 (1.2)	79 (7.2)	78 (6.9)	36 (3.4)	42 (3.9)	23 (2.1)
Women	23 (1.5)	28 (1.9)	100 (6.5)	40 (2.8)	22 (1.5)	3 (0.2)	56 (3.6)

	Major ECG abnormalities		Minor ECG abnormalities				
	T-wave abnormalities	ECG suggestive of cardiomyopathy	LVH	Sinus bradycardia	1st degree atrioventricular conduction disturbances	Mild ventricular conduction disturbances[a]	Mildly prolonged QTc (Bazett) interval[b]
African Surinamese							
Men	50 (3.5)	38 (2.7)	288 (20.3)	124 (8.8)	137 (9.5)	62 (4.3)	33 (2.3)
Women	47 (2.1)	41 (1.9)	340 (15.1)	63 (2.8)	102 (4.5)	16 (0.7)	94 (4.2)
Ghanaian							
Men	40 (4.3)	11 (1.2)	293 (35.5)	41 (5.4)	103 (12.9)	22 (2.5)	9 (1.1)
Women	32 (2.7)	25 (2.1)	266 (21.3)	42 (3.4)	79 (6.1)	7 (0.6)	29 (2.5)
Turkish							
Men	13 (0.9)	10 (0.7)	87 (6.0)	89 (6.2)	55 (3.8)	118 (8.2)	37 (2.5)
Women	11 (0.7)	7 (0.4)	58 (3.3)	36 (2.0)	23 (1.3)	23 (1.4)	69 (3.9)
Moroccan							
Men	6 (0.4)	8 (0.5)	160 (11.5)	122 (9.1)	80 (5.3)	111 (7.8)	14 (0.9)
Women	12 (0.6)	8 (0.4)	108 (4.9)	74 (3.2)	46 (2.1)	29 (1.3)	76 (3.6)

Data are reported in n (%). ECG, electrocardiogram.

[a] 110≤QRS<120ms; [b] men: QTc >450ms, women: QTc ≥460ms.

Appendix Table 4b. Age-adjusted prevalence of less common ECG abnormalities (for majors: prevalence <1% in total study population; for minors: abnormalities not in top 5 with highest prevalence in total study population), shown by major and minor ECG abnormality categories, by sex, ordered by highest to lowest overall prevalence

	**Men**	**Women**
**Major ECG abnormalities**		
Microvoltages	38 (0.5)	138 (1.2)
Right bundle branch block (RBBB)	117 (1.4)	38 (0.3)
Severely prolonged QTc (Bazett) interval[a]	59 (0.7)	91 (0.8)
Nonspecific ventricular conduction disturbances[b]	87 (1.1)	10 (0.1)
Pathological Q waves	50 (0.6)	32 (0.3)
Left bundle branch block (LBBB)	20 (0.2)	36 (0.3)
Ventricular preexcitation	27 (0.3)	16 (0.1)
Extreme axis deviation	15 (0.2)	7 (0.06)
Atrial fibrillation or flutter	8 (0.1)	5 (0.05)
Miscellaneous[c]	7 (0.08)	2 (0.02)
Severely shortened QTc (Bazett) interval[d]	3 (0.04)	2 (0.02)
Ventricular rhythm	2 (0.02)	1 (0.01)
2nd degree atrioventricular conduction disturbances	3 (0.04)	0
Sinus bradycardia + idioventricular rhythm	2 (0.02)	0
3rd degree atrioventricular conduction disturbances	0	0
**Minor ECG abnormalities**		
Left axis deviation	371 (4.5)	207 (1.9)
Right axis deviation	222 (2.8)	120 (1.1)
Possible pathological Q waves	172 (2.1)	111 (1.0)
Frequent PACs and/or PVCs	99 (1.2)	118 (1.1)
Atrial rhythm	79 (1.0)	130 (1.1)
Mildly shortened QTc (Bazett) interval[e]	1.7 (1.4)	100 (0.9)

	**Men**	**Women**
Left anterior fascicular block (LAFB)	61 (0.7)	32 (0.3)
Sinus tachycardia	23 (0.3)	37 (0.3)
Atrial abnormalities	32 (0.4)	27 (0.2)
Indeterminate heart axis	9 (0.1)	4 (0.03)

Data are reported in n (%).

ECG, electrocardiogram; PAC, premature atrial contraction; PVC, premature ventricular contraction.

[a] men: QTc >470ms, women: QTc ≥480ms;

[b] QRS duration ≥120ms.

[c] Including ECG suggestive of Brugada syndrome.

[d] men: QTc <330ms, women: QTc <340ms.

[e] men: QTc <360ms, women: QTc <370ms
