# Supplementary figures and images for: Do sex differences in the prevalence of ECG abnormalities vary across ethnic groups living in the Netherlands? A cross-sectional analysis of the population-based HELIUS study
Source: BMJ Open. 2020 Sep 3;10(9):e039091. doi: 10.1136/bmjopen-2020-039091 (PMC7473628; doi:10.1136/bmjopen-2020-039091)

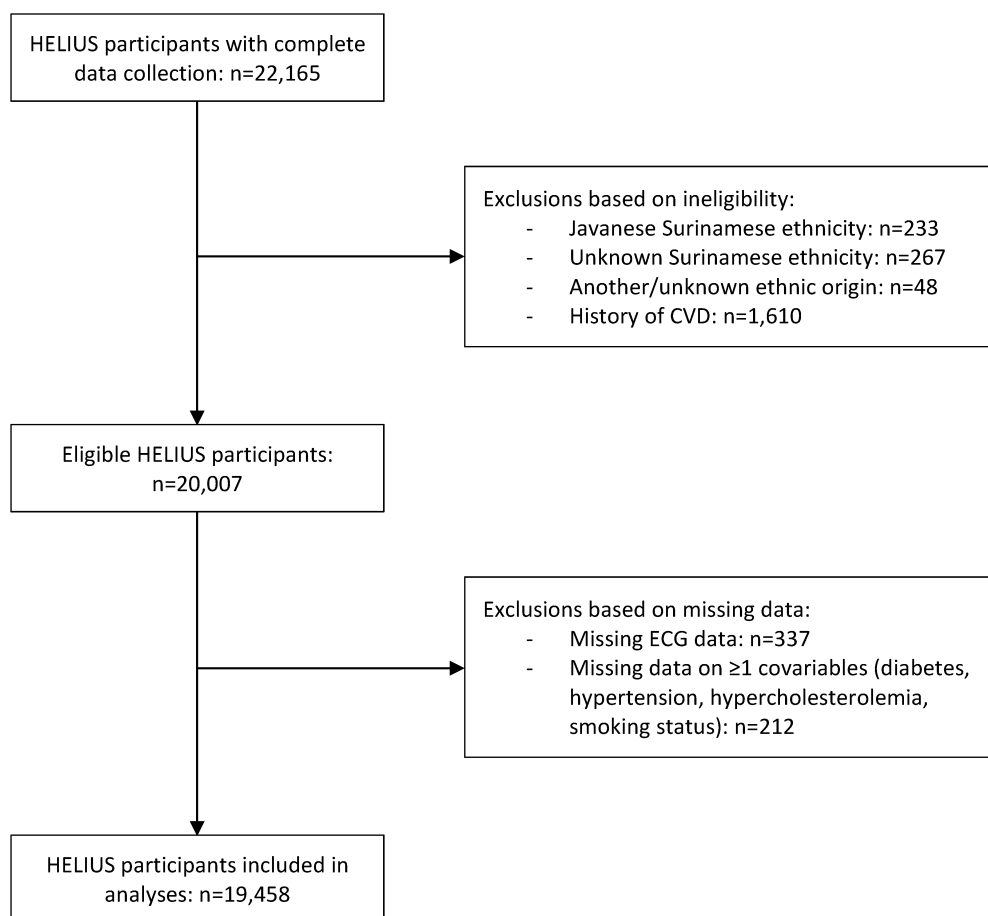

**Appendix Figure 1.** Flow diagram of the study population

Supplement: Supplementary data [file bmjopen-2020-039091supp001.pdf]
