# Supplementary material for: Do sex differences in the prevalence of ECG abnormalities vary across ethnic groups living in the Netherlands? A cross-sectional analysis of the population-based HELIUS study
Source: BMJ Open. 2020 Sep 3;10(9):e039091. doi: 10.1136/bmjopen-2020-039091 (PMC7473628; doi:10.1136/bmjopen-2020-039091)
Supplement: Author's manuscript [file bmjopen-2020-039091.draft_revisions.pdf]

BMJ Open is committed to open peer review. As part of this commitment we make the peer review history of every article we publish publicly available.

When an article is published we post the peer reviewers' comments and the authors' responses online. We also post the versions of the paper that were used during peer review. These are the versions that the peer review comments apply to.

The versions of the paper that follow are the versions that were submitted during the peer review process. They are not the versions of record or the final published versions. They should not be cited or distributed as the published version of this manuscript.

BMJ Open is an open access journal and the full, final, typeset and author-corrected version of record of the manuscript is available on our site with no access controls, subscription charges or pay-per-view fees (http://bmjopen.bmj.com).

If you have any questions on BMJ Open's open peer review process please email info.bmjopen@bmj.com

BMJ Open

Sex differences in the prevalence of electrocardiographic abnormalities across ethnic groups: findings from the population-based HELIUS study

Journal:	*BMJ Open*
Manuscript ID	bmjopen-2020-039091
Article Type:	Original research
Date Submitted by the Author:	06-Apr-2020
Complete List of Authors:	Bolijn, Renee; Amsterdam UMC, Public Health<br>ter Haar, Cato; Amsterdam UMC, Cardiology; Leiden University Medical Center, Cardiology<br>Harskamp, Ralf; Amsterdam UMC, Department of General Practice<br>Tan, Hanno; Amsterdam UMC, Cardiology<br>Kors, Jan; Erasmus Medical Center<br>Postema, Pieter; Amsterdam UMC, Clinical and Experimental Cardiology<br>Snijder, Marieke; Amsterdam UMC, Public Health; Amsterdam UMC, Clinical Epidemiology, Biostatistics and Bioinformatics<br>Peters, Ron; Amsterdam UMC, Cardiology<br>Kunst, Anton; Amsterdam UMC, Public Health<br>van Valkengoed, Irene; Amsterdam UMC, Public Health
Keywords:	Cardiac Epidemiology < CARDIOLOGY, Pacing & electrophysiology < CARDIOLOGY, EPIDEMIOLOGY, PUBLIC HEALTH

SCHOLARONE™
Manuscripts

I, the Submitting Author has the right to grant and does grant on behalf of all authors of the Work (as defined in the below author licence), an exclusive licence and/or a non-exclusive licence for contributions from authors who are: i) UK Crown employees; ii) where BMJ has agreed a CC-BY licence shall apply, and/or iii) in accordance with the terms applicable for US Federal Government officers or employees acting as part of their official duties; on a worldwide, perpetual, irrevocable, royalty-free basis to BMJ Publishing Group Ltd ("BMJ") its licensees and where the relevant Journal is co-owned by BMJ to the co-owners of the Journal, to publish the Work in this journal and any other BMJ products and to exploit all rights, as set out in our licence.

The Submitting Author accepts and understands that any supply made under these terms is made by BMJ to the Submitting Author unless you are acting as an employee on behalf of your employer or a postgraduate student of an affiliated institution which is paying any applicable article publishing charge ("APC") for Open Access articles. Where the Submitting Author wishes to make the Work available on an Open Access basis (and intends to pay the relevant APC), the terms of reuse of such Open Access shall be governed by a Creative Commons licence – details of these licences and which Creative Commons licence will apply to this Work are set out in our licence referred to above.

Other than as permitted in any relevant BMJ Author's Self Archiving Policies, I confirm this Work has not been accepted for publication elsewhere, is not being considered for publication elsewhere and does not duplicate material already published. I confirm all authors consent to publication of this Work and authorise the granting of this licence.

For peer review only

Sex differences in the prevalence of electrocardiographic abnormalities across ethnic groups: findings from the population-based HELIUS study

Renee Bolijn[1], C. Cato ter Haar[2,3], Ralf E. Harskamp[4], Hanno L. Tan[2], Jan A. Kors[5], Pieter G. Postema[6], Marieke B. Snijder[1,7], Ron J.G. Peters[2], Anton E. Kunst[1], Irene G.M. van Valkengoed[1]

[1] *Amsterdam UMC, University of Amsterdam, Department of Public Health, Amsterdam Public Health research institute, Meibergdreef 9, Amsterdam, The Netherlands.*

[2] *Amsterdam UMC, University of Amsterdam, Department of Cardiology, Amsterdam Cardiovascular Sciences, Meibergdreef 9, Amsterdam, The Netherlands.*

[3] *Leiden University Medical Center, Heart-Lung Center, Department of Cardiology, Leiden, The Netherlands.*

[4] *Amsterdam UMC, University of Amsterdam, Department of General Practice, Amsterdam Public Health research institute, Meibergdreef 9, Amsterdam, The Netherlands.*

[5] *Department of Medical Informatics, Erasmus University Medical Center, Rotterdam, The Netherlands.*

[6] *Amsterdam UMC, University of Amsterdam, Department of Clinical and Experimental Cardiology, Amsterdam Cardiovascular Sciences, Meibergdreef 9, Amsterdam, The Netherlands.*

[7] *Amsterdam UMC, University of Amsterdam, Department of Clinical Epidemiology, Biostatistics and Bioinformatics, Amsterdam Public Health research institute, Meibergdreef 9, Amsterdam, The Netherlands*

Corresponding author: Renee Bolijn, Amsterdam UMC, University of Amsterdam, Department of Public Health, Meibergdreef 9, 1105 AZ Amsterdam, The Netherlands. E-mail: r.bolijn@amsterdamumc.nl. Phone: +31 20 5664649

For peer review only - http://bmjopen.bmj.com/site/about/guidelines.xhtml

Word count: 3392

Keywords: ECG abnormalities, sex differences, ethnic minorities, cardiovascular disease prevention, HELIUS study

For peer review only

For peer review only - http://bmjopen.bmj.com/site/about/guidelines.xhtml

ABSTRACT

Objectives Major electrocardiographic (ECG) abnormalities have been associated with increased risk of cardiovascular disease (CVD) burden in asymptomatic populations. However, sex differences in occurrence of major ECG abnormalities have been poorly studied, particularly across ethnic groups. The objectives were to investigate 1) sex differences in the prevalence of major and, as a secondary outcome, minor ECG abnormalities, 2) whether patterns of sex differences varied across ethnic groups, by age, and 3) to what extent conventional cardiovascular risk factors contributed to observed sex differences.

Design Cross-sectional analysis of population-based study.

Setting Multi-ethnic, population-based Healthy Life in an Urban Setting (HELIUS) cohort, Amsterdam, the Netherlands.

Participants 8,089 men and 11,369 women of Dutch, South-Asian Surinamese, African Surinamese, Ghanaian, Turkish, and Moroccan origin aged 18-70 years without CVD.

Outcome measures Age-adjusted and multivariable logistic regression analyses were performed to study sex differences in prevalence of major and, as secondary outcome, minor ECG abnormalities in the overall population, across ethnic groups, and across age-groups (18-35, 36-50, and >50 years).

Results Major and minor ECG abnormalities were less prevalent in women than men (4.6% versus 6.6%, and 23.8% versus 39.8%, respectively). After adjustment for conventional risk factors, sex differences in major abnormalities were smaller in ethnic minority groups (odds ratios [OR] ranged from 0.61 in Moroccans to 1.32 in South-Asian Surinamese) than in the Dutch (OR 0.49; 95% confidence interval [CI] 0.36-0.65). Only in South-Asian Surinamese, women did not have a lower odds than men (OR 1.32; 95% CI 0.96-1.84). The pattern of smaller sex differences in ethnic minority groups was more pronounced in older than in younger age-groups.

For peer review only - http://bmjopen.bmj.com/site/about/guidelines.xhtml

Conclusions The prevalence of major ECG abnormalities was lower in women than men. However, sex differences were less apparent in ethnic minority groups, particularly in older age-groups. Sex differences were not explained by conventional risk factors.

For peer review only

Strengths and limitations of this study

- Participants were sampled from the municipality registry and reflect a general population sample of adults of the major ethnic groups living in Amsterdam.
- Large sample sizes permit the study of sex differences within each ethnic group, including across age strata.
- Single electrocardiographic (ECG) measurements of 10 seconds without additional imaging techniques (e.g., echocardiography) may be suboptimal for the measurement of ECG abnormalities.
- The classification of 'major' and 'minor' ECG abnormalities may depend on criteria used, which may affect reported prevalence estimates.

For peer review only

For peer review only - http://bmjopen.bmj.com/site/about/guidelines.xhtml

INTRODUCTION

The resting electrocardiogram (ECG) is an essential diagnostic instrument in patients with symptoms suggestive of cardiovascular disease (CVD).[1] Previous studies show that the occurrence of major ECG abnormalities is also associated with increased risk of CVD morbidity[2,3] and mortality[3,4] in asymptomatic populations. However, differences in the occurrence of major ECG abnormalities in men and women have been poorly studied. Insights in these sex differences may help to identify subpopulations with a future CVD burden and thus aid targeted (preventive) therapy.

Although studies have described the prevalence of major ECG abnormalities in men and women from diverse populations,[5-11] only three studied sex differences specifically.[5-7] Whether the occurrence of ECG abnormalities differs by sex, independently of cardiovascular risk factors, is a topic of ongoing debate. Two studies suggested that the composite of major ECG abnormalities (e.g., atrial fibrillation, Q-wave or T-wave abnormalities) is more prevalent among men than women,[5,6] while another observed no sex differences.[7]

Differences in the occurrence of ECG abnormalities have been observed between ethnic groups living in similar contexts.[6,8,12,13] However, in Europe, the prevalence of major ECG abnormalities among ethnic minority populations at high risk for CVD, such as men and women of South-Asian origin,[14] is unknown. Additionally, it is unknown to what extent major ECG abnormalities vary between men and women across ethnic groups.

The prevalence of ECG abnormalities tends to increase with increasing age.[5] As larger sex differences in occurrence of CVD have been found in younger age-groups compared to older age-groups,[15] sex differences in prevalence of ECG abnormalities may also vary by age.

For peer review only

For peer review only - http://bmjopen.bmj.com/site/about/guidelines.xhtml

In this study, we investigated sex differences in the prevalence of major and, as a secondary outcome, minor ECG abnormalities, in a 18-70 year-old multi-ethnic population living in Amsterdam, the Netherlands. We assessed whether patterns of sex differences varied across ethnic groups, overall and by age, and to what extent conventional cardiovascular risk factors contributed to observed sex differences, overall and within subgroups.

For peer review only

For peer review only - http://bmjopen.bmj.com/site/about/guidelines.xhtml

METHODS

We used baseline data from the HEalthy LIfe in an Urban Setting (HELIUS) study, a multi-ethnic cohort study conducted in Amsterdam, the Netherlands.[16] Between 2011 and 2015, baseline data were collected among participants of Dutch, South-Asian Surinamese, African Surinamese, Ghanaian, Moroccan, and Turkish origin aged 18-70 years living in Amsterdam. Participants were randomly, stratified by ethnicity, sampled from the municipality registry. Data were obtained by questionnaire and physical examinations (including biological samples). The HELIUS study has been approved by the AMC Ethical Review Board. All participants provided written informed consent.

ECG measurements

Standard 12-lead ECGs were recorded in supine position with a GE MAC5500 electrocardiograph at 500 samples/sec and analysed using the Modular ECG Analysis System (MEANS).[17] The measurement of ECG abnormalities has been described in detail elsewhere.[18] Briefly, ECG abnormalities were assessed by combining ECG diagnoses of the MEANS programs with Minnesota coding, Marquette 12SL ECG analysis software, and a cardiologist's interpretation. In case of discrepancies, ECGs were double checked. We classified ECG abnormalities into major and minor ECG abnormalities (Appendix Table 1).

Ethnicity

Ethnicity was defined by the individual's country of birth combined with the parental countries of birth.[19] Surinamese participants were further classified according to self-reported ethnic origin into "African", "South-Asian", "Javanese", or "other".

Covariables

For peer review only

For peer review only - http://bmjopen.bmj.com/site/about/guidelines.xhtml

Family history of CVD was defined by a self-reported CVD diagnosis among first degree family members aged <60 years. Smoking was classified as current, past, or never smoker. For current smokers, the number of pack-years of smoking was calculated by multiplying the number of packs (containing 20 cigarettes or equivalent rates for cigars and pipe tobacco) smoked a day by the number of years smoked. Physical activity was defined as achieving ≥30 minutes of moderate- or high-intensity activity per day on ≥5 days per week.[20] Alcohol consumption (on average in the last 12 months) was classified as: none or low (men: 0-4; women: 0-2 beverages/week), moderate (men: 5-14; women: 3-7 beverages/week), and high (men: >14; women: >7 beverages/week).

Body mass index (BMI) was calculated in duplicate as weight (kg) divided by height squared (m^2). Blood pressure (BP) was measured in duplicate using a validated automated digital BP device (WatchBP Home; Microlife AG) in a seated position after ≥5 minutes of rest. Hypertension was defined as systolic BP ≥140 mm Hg, diastolic BP ≥90 mm Hg, use of antihypertensive medication treatment, and/or self-reported hypertension.

Fasting blood samples were drawn to determine creatinine, lipid, and glucose concentrations (details on these measurements have been described elsewhere).[21] Chronic kidney disease (CKD) risk was categorized according to the risk of progression of kidney disease based on estimated Glomerular Filtration Rate and albuminuria levels:[22] (1) low, (2) moderately increased, (3) high and very high risk. Hypercholesterolemia was defined as total cholesterol ≥5.0 mmol/l, high-density lipoprotein cholesterol <1.0 mmol/l (men) or <1.2 mmol/l (women), low-density lipoprotein cholesterol ≥3.0 mmol/l (Friedewald formula[23]), triglycerides ≥1.7 mmol/l, use of lipid-lowering medication, and/or self-reported hypercholesterolemia. Participants were considered to have diabetes in case of a fasting glucose ≥7.0 mmol/l, use of glucose-lowering medication and/or if they reported to be diagnosed with diabetes by a doctor.

For peer review only - http://bmjopen.bmj.com/site/about/guidelines.xhtml

Study population

Baseline data were available for 22,165 participants. We excluded those of Javanese Surinamese (n=233), unknown Surinamese (n=267) origin, and with another/unknown ethnic origin (n=48). Next, we excluded participants with a history of CVD (n=1,610; based on self-reported prior myocardial infarction, cerebrovascular accident (CVA), angioplasty or bypass surgery (on heart or legs), use of antiplatelet drugs (Anatomical Therapeutic Chemical [ATC] code B01AC), use of oral anticoagulants (ATC codes B01AA, B01AE, B01AF), use of antiarrhythmic agents (ATC-codes C01A, C01B, C07AA07, C08D), or paced rhythms). Finally, we excluded participants with missing ECG data (n=337) or with missing data on ≥1 covariables (n=212), resulting in a study population of 19,458 participants.

Statistical analyses

Baseline characteristics were expressed as means (standard deviations [SD]) or frequencies (percentages) by sex in the total population and per ethnic group. The age-adjusted prevalence of any major ECG abnormality, any minor ECG abnormality, and a selection of common ECG abnormalities (i.e., major ECG abnormalities with a prevalence of ≥1% and the top 5 most prevalent minor ECG abnormalities) was calculated by sex, in the total population and by ethnicity, using the study population as the standard. For reference, the overall prevalence of less common ECG abnormalities is also provided, but only by sex in the total population. The prevalence of any major ECG abnormalities was also calculated by age-groups (i.e., 18-35, 36-50, and >50 years based on tertiles of the age distribution in the total population) for all ethnic groups.

We performed logistic regression analyses with hierarchal models to examine sex differences in prevalence of any major ECG abnormalities and any minor ECG abnormalities, adjusted for age and ethnicity (model 1), and additionally for hypertension, hypercholesterolemia, diabetes, and smoking

For peer review only - http://bmjopen.bmj.com/site/about/guidelines.xhtml

status (model 2) to determine to what extent conventional cardiovascular risk factors contributed to observed differences. We also examined the additional contribution of other well-known cardiovascular risk factors, i.e., family history of CVD and CKD risk (model 3) and BMI, alcohol consumption, and physical activity (model 4). To study whether the sex differences varied between ethnic groups (i.e., effect modification), a statistical interaction term for sex and ethnicity on a multiplicative scale was added. Then, the main analyses (model 2 with interaction term) for major ECG abnormalities were repeated stratified by age-groups (18-35, 36-50, and >50 years) to examine the consistency of sex differences across ethnic groups among age-groups. All statistical analyses were performed in R studio version 1.1.453.[24] *p*-values <0.05 were regarded as statistically significant.

Sensitivity analyses

We repeated the main analyses excluding obese participants (BMI >30), since obesity may influence the accuracy of ECG measurements.[25] Furthermore, use of psychotropic medication may induce alterations of the ECG resulting in ECG abnormalities (e.g., QT prolongation).[26] Therefore, we repeated the analyses excluding participants with current use of psychotropic medication. Finally, we repeated the analyses using number of pack-years of smoking instead of smoking status, to examine whether the scale of the variables (numeric versus categorical) altered the results.

Patient and public involvement

There was no specific patient or public involvement in the development of the research questions, outcome measures, study design, and recruitment/conduct of the present study. However, for the core HELIUS study, several supportive measures were taken to enhance the enrolment of ethnic minority groups. For example, ethnic-specific communication strategies were used, such as working with faith communities (churches and mosques) and endorsement from local key figures. Understandability of and time to complete the questionnaire were also enquired among participants.

For peer review only - http://bmjopen.bmj.com/site/about/guidelines.xhtml

In addition, the present study is part of a larger project on sex and gender differences in CVD risk. As part of this project, interviews and a short survey on research priority setting according to CVD patients and persons at increased CVD risk were conducted. The present study aligns with our findings from these interviews and survey that more research on sex and gender differences in CVD was perceived as relevant by the target group.

For peer review only

For peer review only - http://bmjopen.bmj.com/site/about/guidelines.xhtml

RESULTS

Mean age was around 43 years (SD 13) in women and 44 years (SD 13) in men (Table 1). More than 20% of both men and women had a family history of CVD. Women were less often current smokers and had fewer mean pack-years of smoking compared to men, while the prevalence of high alcohol consumption was similar among men and women. Women had a higher mean BMI and were less physically active. Hypertension, hypercholesterolemia, and diabetes were less prevalent among women than men, while high CKD risk was equally prevalent among men and women. Women more often used psychotropic medication than men. These patterns in baseline characteristics differed across ethnic groups (Appendix Table 2).

Table 1. Baseline characteristics of 19,458 men and women with ECG measurements

	**Men (n=8,089)**	**Women (n=11,369)**
Age (years)	43.8 (13.0)	43.1 (13.0)
Ethnicity		
Dutch	1,873 (23.2)	2,293 (20.2)
South-Asian Surinamese	1,125 (13.9)	1,464 (12.9)
African Surinamese	1,411 (17.4)	2,266 (19.9)
Ghanaian	822 (10.2)	1,321 (11.6)
Turkish	1,451 (17.9)	1,769 (15.6)
Moroccan	1,407 (17.4)	2,256 (19.8)
Family history of CVD (missing: n=217)	1,637 (20.4)	2,611 (23.3)
Smoker		
Current	2,539 (31.4)	2,032 (17.9)
Past	2,021 (25.0)	1,753 (15.4)
Never	3,529 (43.6)	7,584 (66.7)
Pack-years of smoking (missing: n=191)	5.4 (16.2)	1.8 (7.3)

For peer review only - http://bmjopen.bmj.com/site/about/guidelines.xhtml

	Men (n=8,089)	Women (n=11,369)
Achieving physical activity norm (missing: n=27)	5,020 (62.2)	5,963 (52.5)
Alcohol consumption (missing: n=115)		
None or low	5,981 (74.4)	8,985 (79.5)
Moderate	1,526 (19.0)	1,549 (13.7)
High	528 (6.6)	774 (6.8)
BMI (kg/m^2; missing: n=15)	26.3 (4.2)	27.5 (5.8)
CKD risk (missing: n=63)		
Low	7,684 (95.4)	10,689 (94.3)
Moderate	304 (3.8)	555 (4.9)
High	68 (0.8)	95 (0.8)
Hypertension	3,026 (37.4)	3,594 (31.6)
Hypercholesterolemia	5,752 (71.1)	7,147 (62.9)
Diabetes	829 (10.2)	952 (8.4)
Use of psychotropic medication (missing: n=4)[a]	397 (4.9)	679 (6.0)

Data are presented as means (standard deviations) or frequencies (percentages).

BMI, body mass index; CKD, chronic kidney disease; CVD, cardiovascular disease.

[a] Anatomical Therapeutic Chemical (ATC) codes: N03AE, N03AF, N03AG, N03AN, N05A, N05BA, N05C, N06A, N06BA, N07B, and R06AD02

Overall, the age-adjusted prevalence of major ECG abnormalities was lower among women (4.6%) compared to men (6.6%; Table 2). In most ethnic groups, women had a lower age-adjusted prevalence (range: 2.9%-6.1%) compared to men (range: 4.7%-7.9%), except in the South-Asian Surinamese (7.2% versus 6.0% respectively). Conventional cardiovascular risk factors and other well-known risk factors did not contribute substantially to the observed sex differences in major ECG abnormalities in the total population and within ethnic groups. For instance, the odds ratio (OR) of having a major ECG abnormality changed from 0.69 (95% confidence interval [CI] 0.61-0.78) to 0.71 (95% CI 0.62-0.81)

For peer review only

among women versus men after adjustment for hypertension, hypercholesterolemia, diabetes, and smoking status, and to 0.67 (95% CI 0.58-0.76) after adjustment for family history of CVD, CKD risk, BMI, alcohol consumption, and physical activity.

There was a general pattern of smaller sex differences in occurrence of major ECG abnormalities in the ethnic minority groups compared to the Dutch (Table 2). The adjusted OR for women versus men varied from 0.49 (95% CI 0.36-0.65) in the Dutch to 0.73 (95% CI 0.53-1.01) in Turkish. Only in the South-Asian Surinamese group, women did not have a lower odds than men (adjusted OR 1.32; 95% CI 0.96-1.84).

For peer review only

Table 2. Number of cases and age-adjusted prevalence of any major ECG abnormality by sex in the total population and by ethnic group, and the odds of major ECG abnormalities in women compared to men, overall and with an interaction term for sex and ethnicity

	**Men**	**Women**	**Model 1**				**Model 2**			
	**(n of cases, %[a])**	**(n of cases, %[a])**	**OR (95% CI)**	***p*-value**	**Ratio of ORs (95% CI)***	***p*-value**	**OR (95% CI)**	***p*-value**	**Ratio of ORs (95% CI)***	***p*-value**
Overall	540 (6.6)	518 (4.6)	0.69 (0.61-0.78)[b]	*<0.001*	NA	NA	0.71 (0.62-0.81)[b]	*<0.001*	NA	NA
Dutch	137 (7.3)	79 (3.6)	0.46 (0.34-0.61)	*<0.001*	Reference	NA	0.49 (0.36-0.65)	*<0.001*	Reference	NA
SA Surinamese	63 (6.0)	110 (7.2)	1.24 (0.90-1.72)	0.19	2.69 (1.75-4.17)	*<0.001*	1.32 (0.96-1.84)	0.09	2.72 (1.76-4.21)	*<0.001*
African Surinamese	107 (7.6)	118 (5.3)	0.68 (0.51-0.89)	*<0.01*	1.47 (0.99-2.18)	0.06	0.68 (0.52-0.90)	*<0.01*	1.40 (0.94-2.09)	0.10
Ghanaian	72 (7.9)	71 (6.1)	0.70 (0.49-0.98)	*0.04*	1.51 (0.97-2.37)	0.07	0.71 (0.51-1.01)	0.055	1.47 (0.94-2.30)	0.09
Turkish	89 (6.1)	77 (4.4)	0.71 (0.52-0.98)	*0.04*	1.55 (1.01-2.37)	*0.045*	0.73 (0.53-1.01)	0.058	1.51 (0.98-2.32)	0.06
Moroccan	72 (4.7)	63 (2.9)	0.59 (0.42-0.84)	*<0.01*	1.29 (0.82-2.02)	0.27	0.61 (0.43-0.87)	*<0.01*	1.26 (0.80-1.99)	0.33

CI, confidence interval; NA, not applicable; OR, odds ratio; SA, South-Asian.

Significant *p*-values ($p<0.05$) are printed in italic.

Model 1: adjusted for age; model 2: adjusted for age, hypertension, hypercholesterolemia, diabetes, and smoking status

[a] Age-adjusted prevalence.

[b] Additionally adjusted for ethnicity.

* Measure of effect modification on multiplicative scale (statistical interaction term).

For peer review only

For peer review only - http://bmjopen.bmj.com/site/about/guidelines.xhtml

In the total population, the most frequently observed major ECG abnormalities were T-wave abnormalities (1.2%), microvoltages (1.2%), and (ECG suggestive of) cardiomyopathy (1.1%) among women and T-wave abnormalities (1.6%), right bundle branch block (RBBB; 1.4%), and cardiomyopathy (1.1%) among men. Among South-Asian Surinamese women, the only group of women with no lower odds than men, microvoltages (2.9%), cardiomyopathy (1.9%), and T-wave abnormalities (1.5%) were the most prevalent major ECG abnormalities. T-wave abnormalities (1.9%), cardiomyopathy (1.2%), and RBBB (1.1%) were the most prevalent major ECG abnormalities among South-Asian Surinamese men.

As expected, the prevalence of major ECG abnormalities was higher in older than younger age-groups in both men and women (Figure 1). The general pattern of smaller sex differences in the ethnic minority groups compared to the Dutch differed across the age strata (Table 3). In the older age groups, the adjusted sex difference in the odds of having a major ECG abnormality appeared less pronounced in ethnic minorities compared to the Dutch, whereas this sex difference appeared more similar across ethnic group in the youngest age group. Whereas women in all ethnic groups had a lower odds compared to men across all age strata, this was only the case in the youngest age-group of South-Asian Surinamese women versus men.

Table 3. The odds of major ECG abnormalities in women compared to men by age-groups, in the total population and with an interaction term for sex and ethnicity

**Aged 18-35 years (n=5870)[a]**	**OR (95% CI)**	***p*-value**	**Ratio of ORs (95% CI)***	***p*-value**
Overall[b]	0.38 (0.27-0.54)	*<0.001*	NA	NA
Dutch	0.30 (0.14-0.60)	*<0.01*	Reference	NA
South-Asian Surinamese	0.48 (0.18-1.18)	0.12	1.58 (0.47-5.11)	0.45
African Surinamese	0.38 (0.12-1.05)	0.07	1.25 (0.34-4.44)	0.73

For peer review only - http://bmjopen.bmj.com/site/about/guidelines.xhtml

Ghanaian	0.48 (0.13-1.77)	0.26	1.60 (0.37-7.07)	0.52
Turkish	0.55 (0.28-1.09)	0.09	1.84 (0.69-5.01)	0.23
Moroccan	0.23 (0.09-0.55)	*<0.01*	0.77 (0.23-2.42)	0.65
**Aged 36-50 years (n=7099)[a]**	**OR (95% CI)**	***p*-value**	**Ratio of ORs (95% CI)***	***p*-value**
Overall[b]	0.89 (0.70-1.12)	0.32	NA	NA
Dutch	0.51 (0.28-0.90)	*0.02*	Reference	NA
South-Asian Surinamese	2.61 (1.39-5.20)	*<0.01*	5.13 (2.19-12.56)	*<0.001*
African Surinamese	0.49 (0.29-0.85)	*0.01*	0.97 (0.44-2.16)	0.94
Ghanaian	0.91 (0.50-1.71)	0.76	1.79 (0.78-4.22)	0.18
Turkish	1.04 (0.64-1.69)	0.87	2.04 (0.97-4.38)	0.06
Moroccan	0.92 (0.51-1.67)	0.79	1.81 (0.80-4.17)	0.16
**Aged >50 years (n=6489)[a]**	**OR (95% CI)**	***p*-value**	**Ratio of ORs (95% CI)***	***p*-value**
Overall[b]	0.74 (0.62-0.89)	*<0.01*	NA	NA
Dutch	0.54 (0.37-0.78)	*<0.01*	Reference	NA
South-Asian Surinamese	1.22 (0.79-1.91)	0.37	2.27 (1.28-4.06)	*<0.01*
African Surinamese	0.84 (0.59-1.18)	0.31	1.55 (0.94-2.59)	0.09
Ghanaian	0.74 (0.46-1.17)	0.20	1.37 (0.75-2.48)	0.30
Turkish	0.55 (0.31-0.96)	*0.04*	1.02 (0.51-2.00)	0.96
Moroccan	0.68 (0.40-1.16)	0.16	1.26 (0.66-2.42)	0.49

CI, confidence interval; NA, not applicable; OR, odds ratio.

[a] Model adjustment: age, hypertension, hypercholesterolemia, diabetes, and smoking status.

[b] These models were also adjusted for ethnicity.

* Measure of effect modification on multiplicative scale (statistical interaction term).

Women had a lower prevalence of minor ECG abnormalities (range: 16.2%-35.6%) compared to men (range: 28.5%-55.7%; Appendix Table 3). Sex differences in minor ECG abnormalities were similar across ethnic groups, and were not influenced by conventional risk factors.

For peer review only - http://bmjopen.bmj.com/site/about/guidelines.xhtml

The prevalence of most common ECG abnormalities was also lower in women than men (Appendix Table 4a). Only mildly prolonged QTc interval was more prevalent in women than in men. Patterns were similar across ethnic groups. The prevalence of most less common ECG abnormalities was also lower in women than women, except for microvoltages, severely prolonged QTc (Bazett) interval, and left bundle branch block (LBBB), atrial rhythm, and sinus tachycardia (Appendix Table 4b).

Sensitivity analyses did not alter our interpretation of findings (data not shown).

For peer review only

DISCUSSION

In our study, women have an overall lower age-adjusted prevalence of major ECG abnormalities than men. Sex differences in the prevalence of major ECG abnormalities are smaller in the ethnic minority groups than in the Dutch, particularly in older age-groups. Differences in conventional cardiovascular risk factors and other well-known risk factors do not contribute substantially to these sex differences.

Our study has limitations. First, the results may be affected by selection bias due to non-response (response rate: 28%). Non-response analyses showed that women were more likely to participate than men, Turks and Moroccans were less likely to participate compared to other ethnic groups, and participants were slightly older than non-participants.[16] However, we were able to include large numbers of both men and women, each ethnic group, and age-group, indicating sufficient representation of all subgroups. This is relevant because previous work has shown that relative differences in CVD risk factors between ethnic groups are similar to other European countries,[27] suggesting that our results are generalizable to other European countries. Second, the definition of prior CVD was not comprehensive, as data on self-reported prior CVD other than myocardial infarction and CVA were lacking. However, we also excluded participants with a prior angioplasty or bypass surgery (on heart or legs), or paced rhythms, and those participants using antiplatelet drugs, oral anticoagulants, or antiarrhythmic agents, and verified that our results were consistent in analyses restricted to those with a favourable cardiovascular risk profile (post-hoc analysis in participants without hypertension, hypercholesterolemia, diabetes, and those not smoking; data not shown). Therefore, it is unlikely that our results were substantially affected by misclassification. Third, single ECG measurements may have been suboptimal for the measurement of ECG abnormalities, potentially affecting the prevalence estimates. Some common expressions of CVD might not always be detectable by a single ECG measurement of 10 seconds, such as paroxysmal atrial fibrillation, and some ECG abnormalities need additional diagnostic measurements. However, 24-hour ECG monitoring with

For peer review only

portable ECG devices and additional imaging techniques (e.g., echocardiography) are often not feasible in population-based studies. Finally, the classification of 'major' and 'minor' ECG abnormalities depends on criteria used, and may be variable given the complexity of detailed ECG interpretation. For instance, the level of severity of some abnormalities may depend on the full clinical assessment or on combinations of abnormalities (e.g., RBBB with left axis deviation).

Similar to previous studies reporting on the prevalence of composite major ECG abnormalities stratified by sex,[5-8,10,11] we observed an overall lower prevalence of major ECG abnormalities in women compared to men in most ethnic groups. Prevalence estimates in both men and women were within the range reported in most previous studies (range: 3.0%-13.2%),[5,6,8,10] except two studies with higher estimates.[7,11] T-wave abnormalities were the most prevalent major ECG abnormalities in both men and women in our study and most previous studies.[5-7,10,11] A much larger heterogeneity has been reported in previous studies in prevalence of minor ECG abnormalities, ranging from 4.5% to 31.6% in women[5,6,8,9,11] and from 7.3% to 45.7% in men.[5,6,8,9,11] Our prevalence was higher compared to most studies, most likely due to differences in the classification of major and minor ECG abnormalities.

The observed sex differences in major CG abnormalities are in line with known differences in cardiovascular pathophysiology and epidemiology of CVD between men and women.[28,29] For instance, men tend to develop coronary artery disease (CAD) earlier than women, resulting in a higher incidence of CAD in men compared to women, in particular at a younger age.[28,29] This age-effect is consistent with our observations across ethnic groups of larger sex differences in prevalence of major ECG abnormalities in the youngest age-group compared to the older age-groups.

For peer review only - http://bmjopen.bmj.com/site/about/guidelines.xhtml

Differential patterning of cardiovascular risk factors did not explain the observed sex differences in prevalence of major ECH abnormalities overall and across ethnic groups. This finding is consistent with two previous studies on sex differences in ECG abnormalities[5,6] but not with another study.[7] Other explanations for the relative cardiovascular advantage of women compared to men at a younger age are still unclear, but may relate to sex hormones, with a prominent role for the protective effects of estrogen in the development of CVD among premenopausal women.[28] Our findings of larger sex differences in prevalence of major ECG abnormalities in the youngest age-group compared to the older age-groups support this hypothesis.

We observed that only South-Asian Surinamese women did not have a lower odds of having a major ECG abnormality compared to South-Asian Surinamese men, which was mainly due to the higher prevalence of major ECG abnormalities among South-Asian Surinamese women compared to other women. South-Asian populations living in Europe are already considered a high-risk population for CVD[14] and our findings may suggest that South-Asian Surinamese women specifically are a target group for CVD prevention strategies. Although women had a consistently lower odds of having a major ECG abnormality than men in all other ethnic groups (except South-Asian Surinamese), Dutch women had a larger cardiovascular advantage than the other women. These findings are in line with a previous study from the USA showing a larger gap between men and women of the white majority population compared to black men and women in CAD mortality.[30] In contrast, a Dutch study on sex disparities in myocardial infarction incidence observed a smaller sex difference in the Dutch majority population compared to minority populations originating from Morocco, South-Asia, and Turkey.[15] Explanations for the discrepancy between this and our study are unclear, but may relate to differences in study populations and exclusion criteria.

For peer review only - http://bmjopen.bmj.com/site/about/guidelines.xhtml

Differential patterning of cardiovascular risk factors did not explain the smaller cardiovascular advantage among minority women compared to Dutch women, suggesting that other factors may be relevant. Psychosocial factors (e.g., discrimination), for instance, may be important risk factors for major ECG abnormalities in some groups of participants, potentially through stress and lifestyle-related factors. For instance, an American study found that current and chronic stress were associated with subclinical atherosclerosis in South-Asian women but not in South-Asian men.[31] Further research needs to confirm whether these psychosocial factors may also explain ethnic-specific variation in sex differences in occurrence of major ECG abnormalities.

The observed sex differences in occurrence of major ECG abnormalities, overall and within subgroups, may also reflect that ECG reference values do not differentiate between men and women (except QTc duration), ethnic groups, or age-groups. Normal values for ECGs may differ for women[32] and non-white groups[33] compared to white men, in whom the ECG reference criteria were developed. This is problematic since subgroups with pathological ECGs and potentially related cardiovascular risk might have been missed, or have a false positive diagnosis. For example, some studies suggest that current ECG criteria for microvoltages may be less valid for women[34] and Asian populations,[35] which may have resulted in an overestimation of the occurrence of microvoltages among South-Asian Surinamese women in our study.

Our results have potential implications. We observed sex differences in ECG abnormalities and identified subpopulations with a relatively high prevalence, e.g., Dutch men, and men and women of South-Asian and African origin. Moreover, we found that these sex differences occurred irrespective of conventional risk factors. Previous studies have suggested that ECG measures may be, in addition to established cardiovascular risk factors, useful for the prediction of future CVD in intermediate and high-risk groups.[36,37] However, evidence is still limited and potential harms of screening are

For peer review only - http://bmjopen.bmj.com/site/about/guidelines.xhtml

unknown. Hence, screening for CVD with ECG is currently not recommended.[38] ECG reference values should be validated in ethnically diverse populations of men and women of different age-groups in order to further investigate the potentially added value of an ECG to cardiovascular risk classification.

For peer review only

For peer review only - http://bmjopen.bmj.com/site/about/guidelines.xhtml

ACKNOWLEDGEMENTS

We are most grateful to the participants of the HELIUS study and the management team, research nurses, interviewers, research assistants and other staff who have taken part in gathering the data of this study.

COMPETING INTERESTS

None declared.

FUNDING

The HELIUS study is conducted by the Academic Medical Center Amsterdam and the Public Health Service of Amsterdam. Both organizations provided core support for HELIUS. The HELIUS study is also funded by the Dutch Heart Foundation, the Netherlands Organization for Health Research and Development (ZonMw), European Union (FP-7), and the European Fund for the Integration of non-EU immigrants (EIF). This work was additionally supported by a grant from the ZonMw Gender and Health Program (grant number 849200008). HLT has received funding from the European Union's Horizon 2020 research and innovation programme under acronym ESCAPE-NET (grant number 733381).

For peer review only

For peer review only - http://bmjopen.bmj.com/site/about/guidelines.xhtml

AUTHORS' CONTRIBUTIONS

RB and IGMvV contributed to the conception and design of the work; CCtH, REH, PGP, and AEK contributed to the design. MBS and RJP contributed to the acquisition of the data; all authors contributed to the analysis and interpretation of the results. RB drafted the manuscript. CCtH, REH, HLT, JAK, PGP, MBS, RJGP, AEK, and IGMvV critically revised the manuscript. All authors gave final approval and agree to be accountable for all aspects of work ensuring integrity and accuracy.

For peer review only

For peer review only - http://bmjopen.bmj.com/site/about/guidelines.xhtml

REFERENCES

[1] Fye WB. A history of the origin, evolution, and impact of electrocardiography. *Am J Cardiol* 1994;73:937-949.

[2] Denes P, Larson JC, Lloyd-Jones DM, et al. Major and minor ECG abnormalities in asymptomatic women and risk of cardiovascular events and mortality. *JAMA* 2007;297:978-985.

[3] Machado DB, Crow RS, Boland LL, et al. Electrocardiographic findings and incident coronary heart disease among participants in the Atherosclerosis Risk in Communities (ARIC) study. *Am J Cardiol* 2006;97:1176-1181.

[4] De Bacquer D, De Backer G, Kornitzer M, et al. Prognostic value of ECG findings for total, cardiovascular disease, and coronary heart disease death in men and women. *Heart* 1998;80:570-577.

[5] De Bacquer D, De Backer G and Kornitzer M. Prevalences of ECG findings in large population based samples of men and women. *Heart* 2000;84:625-633.

[6] Vitelli LL, Crow RS, Shahar E, et al. Electrocardiographic findings in a healthy biracial population. *Am J Cardiol* 1998;81:453-459.

[7] Prineas RJ, Le A, Soliman EZ, et al. United States national prevalence of electrocardiographic abnormalities in black and white middle-age (45-to 64-year) and older (≥65-year) adults (from the Reasons for Geographic and Racial Differences in Stroke Study). *Am J Cardiol* 2012;109:1223-1228.

[8] Walsh III JA, Prineas R, Daviglus ML, et al. Prevalence of electrocardiographic abnormalities in a middle-aged, biracial population: Coronary Artery Risk Development in Young Adults study. *J Electrocardiol* 2010;43:385.e1-385.e9.

[9] Liao YL, Liu KA, Dyer A, et al. Major and minor electrocardiographic abnormalities and risk of death from coronary heart disease, cardiovascular diseases and all causes in men and women. *J Am Coll Cardiol* 1988;12:1494-1500.

For peer review only - http://bmjopen.bmj.com/site/about/guidelines.xhtml

[10] Pinto-Filho MM, Brant LC, Foppa M, et al. Major electrocardiographic abnormalities according to the Minnesota Coding System among Brazilian adults (from the ELSA-Brasil Cohort Study). *Am J Cardiol* 2017;119:2081-2087.

[11] Wu C-C, Yeh W-T, Crow RS, et al. Comparison of electrocardiographic findings and associated risk factors between Taiwan Chinese and US White adults. *Int J Cardiol* 2008;128:224-231.

[12] Perez MV, Yaw TS, Myers J, et al. Prognostic value of the computerized ECG in Hispanics. *Clin Cardiol* 2007;30:189-194.

[13] Santhanakrishnan R, Wang N, Larson MG, et al. Racial differences in electrocardiographic characteristics and prognostic significance in Whites versus Asians. *J Am Heart Assoc* 2016;5:e002956.

[14] Cainzos-Achirica M, Fedeli U, Sattar N, et al. Epidemiology, risk factors, and opportunities for prevention of cardiovascular disease in individuals of South Asian ethnicity living in Europe. *Atherosclerosis* 2019;286:105-113.

[15] van Oeffelen AA, Vaartjes I, Stronks K, et al. Sex disparities in acute myocardial infarction incidence: Do ethnic minority groups differ from the majority population? *Eur J Prev Cardiol* 2015;22:180-188.

[16] Snijder MB, Galenkamp H, Prins M, et al. Cohort profile: the Healthy Life in an Urban Setting (HELIUS) study in Amsterdam, the Netherlands. *BMJ Open* 2017;7:e017873.

[17] van Bemmel J, Kors J and Van Herpen G. Methodology of the modular ECG analysis system MEANS. *Methods Inf Med* 1990;29:346-353.

[18] ter Haar C, Kors J, Peters R, et al. The prevalence of electrocardiograms exceeding thresholds for ST-elevation myocardial infarction in apparently healthy individuals: the role of ethnicity. *J Am Heart Assoc* 2020:Accepted for publication.

[19] Stronks K, Kulu-Glasgow I and Agyemang C. The utility of 'country of birth' for the classification of ethnic groups in health research: the Dutch experience. *Ethn Health* 2009;14:255-269.

For peer review only

For peer review only - http://bmjopen.bmj.com/site/about/guidelines.xhtml

[20] Wendel-Vos GW, Schuit AJ, Saris WH, et al. Reproducibility and relative validity of the short questionnaire to assess health-enhancing physical activity. *J Clin Epidemiol* 2003;56:1163-1169.

[21] Agyemang C, Snijder MB, Adjei DN, et al. Ethnic disparities in CKD in the Netherlands: the HEalthy LIfe in an Urban Setting (HELIUS) study. *Am J Kidney Dis* 2016;67:391-399.

[22] Levin A, Stevens PE, Bilous RW, et al. Kidney Disease: Improving Global Outcomes (KDIGO) CKD Work Group. KDIGO 2012 clinical practice guideline for the evaluation and management of chronic kidney disease. *Kidney Int Suppl* 2013;3:1-150.

[23] Friedewald WT, Levy RI and Fredrickson DS. Estimation of the concentration of low-density lipoprotein cholesterol in plasma, without use of the preparative ultracentrifuge. *Clin Chem* 1972;18:499-502.

[24] RStudio Team. RStudio: Integrated Development for R. Boston, MA: RStudio, Inc., 2016.

[25] Abergel E, Tase M, Menard J, et al. Influence of obesity on the diagnostic value of electrocardiographic criteria for detecting left ventricular hypertrophy. *Am J Cardiol* 1996;77:739-744.

[26] Vlachos K, Georgopoulos S, Efremidis M, et al. An update on risk factors for drug-induced arrhythmias. *Expert Rev Clin Pharmacol* 2016;9:117-127.

[27] Agyemang C, Kunst AE, Bhopal R, et al. Brief report: diabetes prevalence in populations of South Asian Indian and African origins: a comparison of England and The Netherlands. *Epidemiology* 2011;22:563-567.

[28] Regitz-Zagrosek V and Kararigas G. Mechanistic pathways of sex differences in cardiovascular disease. *Physiol Rev* 2016;97:1-37.

[29] Mosca L, Barrett-Connor E and Kass Wenger N. Sex/gender differences in cardiovascular disease prevention: what a difference a decade makes. *Circulation* 2011;124:2145-2154.

[30] Ho JE, Paultre F and Mosca L. The gender gap in coronary heart disease mortality: is there a difference between blacks and whites? *J Womens Health* 2005;14:117-127.

For peer review only - http://bmjopen.bmj.com/site/about/guidelines.xhtml

[31] Shah BM, Shah S, Kandula NR, et al. Psychosocial factors associated with subclinical atherosclerosis in South Asians: the MASALA study. *J Immigr Minor Health* 2016;18:1317-1327.

[32] Rijnbeek PR, Van Herpen G, Bots ML, et al. Normal values of the electrocardiogram for ages 16-90 years. *J Electrocardiol* 2014;47:914-921.

[33] Macfarlane P, Katibi I, Hamde S, et al. Racial differences in the ECG-selected aspects. *J Electrocardiol* 2014;47:809-814.

[34] Clark E, Devine B and Macfarlane P. Age and sex dependent criteria for lower limits of QRS voltages. *2006 Computers in Cardiology*. Valencia, Spain: IEEE, 17-20 September 2006, p. 17-20.

[35] Yang T-F and Macfarlane PW. Comparison of the derived vectorcardiogram in apparently healthy whites and Chinese. *Chest* 1994;106:1014-1020.

[36] Badheka AO, Patel N, Tuliani TA, et al. Electrocardiographic abnormalities and reclassification of cardiovascular risk: insights from NHANES-III. *Am J Med* 2013;126:319-326.

[37] Auer R, Bauer DC, Marques-Vidal P, et al. Association of major and minor ECG abnormalities with coronary heart disease events. *JAMA* 2012;307:1497-1505.

[38] Jonas DE, Reddy S, Middleton JC, et al. Screening for cardiovascular disease risk with resting or exercise electrocardiography: evidence report and systematic review for the US Preventive Services Task Force. *JAMA* 2018;319:2315-2328.

For peer review only

For peer review only - http://bmjopen.bmj.com/site/about/guidelines.xhtml

FIGURE LEGEND

Figure 1. Prevalence of any major ECG abnormalities in men and women by age-groups and ethnicity.

For peer review only

For peer review only - http://bmjopen.bmj.com/site/about/guidelines.xhtml

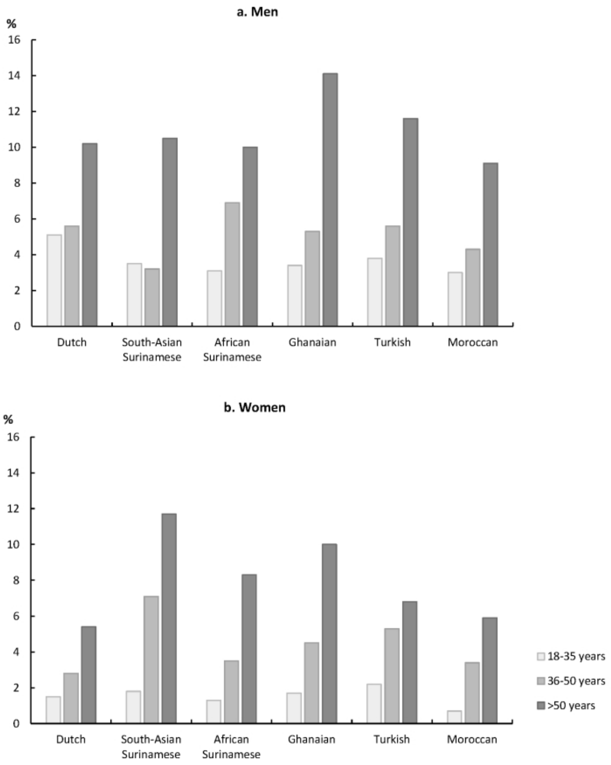

218x309mm (300 x 300 DPI)

Appendix Table 1. Classification of major and minor ECG abnormalities

**Major ECG abnormalities**
Atrial fibrillation or flutter
Ventricular rhythm
Sinus bradycardia + idioventricular rhythm
2nd degree atrioventricular conduction disturbances
3rd degree atrioventricular conduction disturbances
Ventricular preexcitation
Left bundle branch block (LBBB)
Right bundle branch block (RBBB)
Nonspecific ventricular conduction disturbances (QRS duration ≥120ms)
Severely prolonged QTc (Bazett) interval (men: QTc >470ms, women: QTc ≥480ms)
Severely shortened QTc (Bazett) interval (men: QTc <330ms, women: QTc <340ms)
Extreme axis deviation
Pathological Q waves
Microvoltages
T-wave abnormalities
ECG suggestive of cardiomyopathy
Miscellaneous (including ECG suggestive of Brugada syndrome)
**Minor ECG abnormalities**
Sinus tachycardia
Sinus bradycardia
Atrial rhythm
Frequent premature atrial contractions (PACs) and/or premature ventricular contractions (PVCs)
Atrial abnormalities
1st degree atrioventricular conduction disturbances
Left anterior fascicular block (LAFB)
Mild (110≤QRS<120ms) ventricular conduction disturbances, including incomplete LBBB and incomplete RBBB

For peer review only

Mildly prolonged QTc (Bazett) interval (men: QTc >450ms, women: QTc ≥460ms)
Mildly shortened QTc (Bazett) interval (men: QTc <360ms, women: QTc <370ms)
Left axis deviation
Right axis deviation
Indeterminate heart axis
Possible pathological Q waves
Left ventricular hypertrophy (LVH)

ECG, electrocardiogram.

For peer review only

Appendix Table 2. Baseline characteristics of 19,548 participants with ECG measurements, by sex and ethnicity

	Dutch		South-Asian Surinamese		African Surinamese	
	**Men (n=1,873)**	**Women (n=2,293)**	**Men (n=1,125)**	**Women (n=1,464)**	**Men (n=1,411)**	**Women (n=2,266)**
Age (years)	45.8 (13.6)	44.8 (14.0)	42.6 (13.2)	45.0 (13.1)	47.2 (12.9)	47.1 (12.3)
Family history of CVD	418 (22.4)	536 (23.5)	399 (35.8)	583 (40.5)	231 (16.5)	473 (21.2)
(missing: n=217*)						
Smoker						
Current	477 (25.5)	532 (23.2)	432 (38.4)	277 (18.9)	597 (42.3)	548 (24.2)
Past	722 (38.5)	842 (36.7)	177 (15.7)	159 (10.9)	291 (20.6)	400 (17.7)
Never	674 (36.0)	919 (40.1)	516 (45.9)	1,028 (70.2)	523 (37.1)	1,318 (58.2)
Pack-years of smoking	3.4 (10.6)	2.9 (9.7)	8.9 (26.9)	1.7 (5.9)	8.3 (20.3)	2.5 (9.8)
(missing: n=191*)						
Achieving physical activity norm	1,365 (72.9)	1,792 (78.2)	645 (57.5)	729 (49.9)	983 (69.8)	1,270 (56.1)
(missing: n=27*)						
Alcohol consumption						
(missing: n=115*)						
None or low	721 (38.6)	774 (33.9)	880 (78.6)	1,261 (86.7)	1,056 (75.6)	1,883 (83.7)
Moderate	884 (47.3)	925 (40.5)	151 (13.5)	149 (10.2)	260 (18.6)	278 (12.4)

For peer review only

High	264 (14.1)	587 (25.7)	89 (7.9)	44 (3.0)	80 (5.7)	89 (4.0)
BMI (kg/m^2; missing: n=15*)	25.0 (3.7)	24.2 (4.3)	25.7 (4.1)	26.4 (5.2)	26.2 (4.3)	28.6 (5.9)
CKD risk (missing: n=63*)						
Low	1,804 (96.8)	2,216 (96.9)	1,046 (93.2)	1,365 (93.5)	1,344 (95.5)	2,139 (94.6)
Moderate	49 (2.6)	64 (2.8)	62 (5.5)	78 (5.3)	53 (3.8)	100 (4.4)
High	10 (0.5)	7 (0.3)	14 (1.2)	17 (1.2)	11 (0.8)	21 (0.9)
Hypertension	618 (33.0)	471 (20.5)	429 (38.1)	527 (36.0)	660 (46.8)	1,072 (47.3)
Hypercholesterolemia	1,314 (70.2)	1,374 (59.9)	934 (83.0)	1,120 (76.5)	889 (63.0)	1,447 (63.9)
Diabetes	76 (4.1)	40 (1.7)	190 (16.9)	209 (14.3)	149 (10.6)	237 (10.5)
Use of psychotropic medication (missing: n=4*)	88 (4.7)	190 (8.3)	57 (5.1)	100 (6.8)	53 (3.8)	105 (4.6)

	**Ghanaian**		**Turkish**		**Moroccan**	
	**Men (n=822)**	**Women (n=1,321)**	**Men (n=1,451)**	**Women (n=1,769)**	**Men (n=1,407)**	**Women (n=2,256)**
Age (years)	46.3 (11.6)	43.0 (10.7)	39.8 (11.9)	39.1 (12.0)	41.6 (12.7)	39.1 (12.8)
Family history of CVD (missing: n=217*)	36 (4.4)	53 (4.1)	382 (26.7)	615 (35.2)	171 (12.3)	351 (15.8)
Smoker						
Current	60 (7.3)	32 (2.4)	602 (41.5)	521 (29.5)	371 (26.4)	122 (5.4)

For peer review only

Past	102 (12.4)	66 (5.0)	354 (24.4)	207 (11.7)	375 (26.7)	79 (3.5)
Never	660 (80.3)	1,223 (92.6)	495 (34.1)	1,041 (58.8)	661 (47.0)	2,055 (91.1)
Pack-years of smoking (missing: n=191*)	0.6 (4.2)	0.2 (2.3)	6.9 (14.8)	2.9 (7.2)	3.8 (9.9)	0.3 (1.9)
Achieving physical activity norm (missing: n=27*)	516 (62.8)	626 (47.4)	720 (49.8)	618 (35.0)	791 (56.4)	928 (41.2)
Alcohol consumption (missing: n=115*)						
None or low	711 (87.6)	1,184 (90.5)	1,291 (89.8)	1,679 (95.3)	1,322 (94.4)	2,204 (98.0)
Moderate	98 (12.1)	103 (7.9)	87 (6.1)	62 (3.5)	46 (3.3)	32 (1.4)
High	3 (0.4)	22 (1.7)	59 (4.1)	20 (1.1)	33 (2.4)	12 (0.5)
BMI (kg/m^2; missing: n=15*)	26.6 (3.6)	29.5 (5.3)	27.7 (4.4)	28.9 (6.5)	26.7 (4.0)	28.0 (5.7)
CKD risk (missing: n=63*)						
Low	770 (94.2)	1,213 (92.2)	1,379 (95.4)	1,645 (93.3)	1,341 (95.7)	2,111 (93.7)
Moderate	37 (4.5)	87 (6.6)	57 (3.9)	106 (6.0)	46 (3.3)	120 (5.3)
High	10 (1.2)	16 (1.2)	9 (0.6)	12 (0.7)	14 (1.0)	22 (1.0)
Hypertension	494 (60.1)	673 (50.9)	427 (29.4)	407 (23.0)	398 (28.3)	444 (19.7)
Hypercholesterolemia	551 (67.0)	792 (60.0)	1,120 (77.2)	1,130 (63.9)	944 (67.1)	1,284 (56.9)

For peer review only

Diabetes	115 (14.0)	110 (8.3)	138 (9.5)	134 (7.6)	161 (11.4)	222 (9.8)
Use of psychotropic medication	24 (2.9)	41 (3.1)	88 (6.1)	139 (7.9)	87 (6.2)	104 (4.6)
(missing: n=4*)						

Data are presented as means (standard deviations) or frequencies (percentages).

BMI, body mass index; CKD, chronic kidney disease; CVD, cardiovascular disease.

* From total study population (n=19,458).

For peer review only

Appendix Table 3. Number of cases and age-adjusted prevalence of any minor ECG abnormality by sex in the total population and by ethnic group, and the odds of minor ECG abnormalities in women compared to men, overall and with an interaction term for sex and ethnicity

	**Men**	**Women**	**Model 1**				**Model 2**			
	**(n of cases, %[a])**	**(n of cases, %[a])**	**OR (95% CI)**	***p*-value**	**Ratio of ORs (95% CI)***	***p*-value**	**OR (95% CI)**	***p*-value**	**Ratio of ORs (95% CI)***	***p*-value**
Overall	3,227 (39.8)	2,684 (23.8)	0.45 (0.42-0.48)[b]	*<0.001*	NA	NA	0.46 (0.43-0.49)[b]	*<0.001*	NA	NA
Dutch	829 (44.3)	636 (27.6)	0.49 (0.43-0.55)	*<0.001*	Reference	NA	0.50 (0.44-0.57)	*<0.001*	Reference	NA
SA Surinamese	316 (28.5)	269 (17.9)	0.56 (0.46-0.67)	*<0.001*	1.15 (0.92-1.44)	0.22	0.57 (0.47-0.68)	*<0.001*	1.13 (0.90-1.42)	0.28
African Surinamese	647 (45.5)	638 (28.4)	0.46 (0.40-0.53)	*<0.001*	0.95 (0.78-1.15)	0.59	0.46 (0.40-0.53)	*<0.001*	0.93 (0.76-1.12)	0.43
Ghanaian	455 (55.7)	452 (35.6)	0.43 (0.36-0.52)	*<0.001*	0.89 (0.72-1.11)	0.31	0.44 (0.36-0.52)	*<0.001*	0.87 (0.70-1.09)	0.22
Turkish	468 (32.4)	283 (16.2)	0.40 (0.34-0.48)	*<0.001*	0.83 (0.67-1.02)	0.08	0.40 (0.34-0.47)	*<0.001*	0.80 (0.64-0.99)	*0.04*
Moroccan	512 (36.5)	406 (18.3)	0.39 (0.34-0.46)	*<0.001*	0.81 (0.66-0.99)	*0.04*	0.39 (0.34-0.46)	*<0.001*	0.78 (0.64-0.96)	*0.02*

CI, confidence interval; NA, not applicable; OR, odds ratio; SA, South-Asian.

Significant *p*-values (p<0.05) are printed in italic.

Model 1: adjusted for age.

Model 2: adjusted for age, hypertension, hypercholesterolemia, diabetes, and smoking status.

[a] Age-adjusted prevalence.

For peer review only

[b] Additionally adjusted for ethnicity.

* Measure of effect modification on multiplicative scale (statistical interaction term).

For peer review only

Appendix Table 4a. Age-adjusted prevalence of a selection of common ECG abnormalities (for majors: prevalence ≥1% in total study population; for minors: top 5 of abnormalities with highest prevalence in total study population), shown by major and minor ECG abnormality categories, by sex and ethnicity, ordered by highest to lowest overall prevalence

	Major ECG abnormalities		Minor ECG abnormalities				
	T-wave abnormalities	ECG suggestive of cardiomyopathy	LVH	Sinus bradycardia	1st degree atrioventricular conduction disturbances	Mild ventricular conduction disturbances[a]	Mildly prolonged QTc (Bazett) interval[b]
All							
Men	133 (1.6)	90 (1.1)	1,088 (13.5)	723 (9.0)	532 (6.5)	609 (7.5)	173 (2.1)
Women	137 (1.2)	124 (1.1)	986 (8.8)	473 (4.2)	344 (3.1)	133 (1.2)	393 (3.5)
Dutch							
Men	7 (0.4)	10 (0.5)	181 (9.8)	269 (14.3)	121 (6.3)	254 (13.6)	57 (3.0)
Women	12 (0.5)	15 (0.6)	114 (5.0)	218 (9.4)	72 (3.1)	55 (2.4)	69 (3.1)
South-Asian Surinamese							
Men	17 (1.9)	13 (1.2)	79 (7.2)	78 (6.9)	36 (3.4)	42 (3.9)	23 (2.1)
Women	23 (1.5)	28 (1.9)	100 (6.5)	40 (2.8)	22 (1.5)	3 (0.2)	56 (3.6)

For peer review only

	Major ECG abnormalities		Minor ECG abnormalities				
	T-wave abnormalities	ECG suggestive of cardiomyopathy	LVH	Sinus bradycardia	1st degree atrioventricular conduction disturbances	Mild ventricular conduction disturbances[a]	Mildly prolonged QTc (Bazett) interval[b]
African Surinamese							
Men	50 (3.5)	38 (2.7)	288 (20.3)	124 (8.8)	137 (9.5)	62 (4.3)	33 (2.3)
Women	47 (2.1)	41 (1.9)	340 (15.1)	63 (2.8)	102 (4.5)	16 (0.7)	94 (4.2)
Ghanaian							
Men	40 (4.3)	11 (1.2)	293 (35.5)	41 (5.4)	103 (12.9)	22 (2.5)	9 (1.1)
Women	32 (2.7)	25 (2.1)	266 (21.3)	42 (3.4)	79 (6.1)	7 (0.6)	29 (2.5)
Turkish							
Men	13 (0.9)	10 (0.7)	87 (6.0)	89 (6.2)	55 (3.8)	118 (8.2)	37 (2.5)
Women	11 (0.7)	7 (0.4)	58 (3.3)	36 (2.0)	23 (1.3)	23 (1.4)	69 (3.9)
Moroccan							
Men	6 (0.4)	8 (0.5)	160 (11.5)	122 (9.1)	80 (5.3)	111 (7.8)	14 (0.9)
Women	12 (0.6)	8 (0.4)	108 (4.9)	74 (3.2)	46 (2.1)	29 (1.3)	76 (3.6)

Data are reported in n (%). ECG, electrocardiogram.

For peer review only

[a] 110≤QRS<120ms; [b] men: QTc >450ms, women: QTc ≥460ms.

For peer review only

Appendix Table 4b. Age-adjusted prevalence of less common ECG abnormalities (for majors: prevalence <1% in total study population; for minors: abnormalities not in top 5 with highest prevalence in total study population), shown by major and minor ECG abnormality categories, by sex, ordered by highest to lowest overall prevalence

	**Men**	**Women**
**Major ECG abnormalities**		
Microvoltages	38 (0.5)	138 (1.2)
Right bundle branch block (RBBB)	117 (1.4)	38 (0.3)
Severely prolonged QTc (Bazett) interval[a]	59 (0.7)	91 (0.8)
Nonspecific ventricular conduction disturbances[b]	87 (1.1)	10 (0.1)
Pathological Q waves	50 (0.6)	32 (0.3)
Left bundle branch block (LBBB)	20 (0.2)	36 (0.3)
Ventricular preexcitation	27 (0.3)	16 (0.1)
Extreme axis deviation	15 (0.2)	7 (0.06)
Atrial fibrillation or flutter	8 (0.1)	5 (0.05)
Miscellaneous[c]	7 (0.08)	2 (0.02)
Severely shortened QTc (Bazett) interval[d]	3 (0.04)	2 (0.02)
Ventricular rhythm	2 (0.02)	1 (0.01)
2nd degree atrioventricular conduction disturbances	3 (0.04)	0
Sinus bradycardia + idioventricular rhythm	2 (0.02)	0
3rd degree atrioventricular conduction disturbances	0	0
**Minor ECG abnormalities**		
Left axis deviation	371 (4.5)	207 (1.9)
Right axis deviation	222 (2.8)	120 (1.1)
Possible pathological Q waves	172 (2.1)	111 (1.0)
Frequent PACs and/or PVCs	99 (1.2)	118 (1.1)
Atrial rhythm	79 (1.0)	130 (1.1)
Mildly shortened QTc (Bazett) interval[e]	1.7 (1.4)	100 (0.9)

For peer review only

	Men	Women
Left anterior fascicular block (LAFB)	61 (0.7)	32 (0.3)
Sinus tachycardia	23 (0.3)	37 (0.3)
Atrial abnormalities	32 (0.4)	27 (0.2)
Indeterminate heart axis	9 (0.1)	4 (0.03)

Data are reported in n (%).

ECG, electrocardiogram; PAC, premature atrial contraction; PVC, premature ventricular contraction.

[a] men: QTc >470ms, women: QTc ≥480ms;

[b] QRS duration ≥120ms.

[c] Including ECG suggestive of Brugada syndrome.

[d] men: QTc <330ms, women: QTc <340ms.

[e] men: QTc <360ms, women: QTc <370ms

For peer review only

STROBE 2007 (v4) Statement—Checklist of items that should be included in reports of *cross-sectional studies*

Section/Topic	Item #	Recommendation	Reported on page #
**Title and abstract**	1	(*a*) Indicate the study's design with a commonly used term in the title or the abstract	1
		(*b*) Provide in the abstract an informative and balanced summary of what was done and what was found	2, 3
**Introduction**			
Background/rationale	2	Explain the scientific background and rationale for the investigation being reported	6
Objectives	3	State specific objectives, including any prespecified hypotheses	7
**Methods**			
Study design	4	Present key elements of study design early in the paper	8
Setting	5	Describe the setting, locations, and relevant dates, including periods of recruitment, exposure, follow-up, and data collection	8
Participants	6	(*a*) Give the eligibility criteria, and the sources and methods of selection of participants	8, 10
Variables	7	Clearly define all outcomes, exposures, predictors, potential confounders, and effect modifiers. Give diagnostic criteria, if applicable	8, 9
Data sources/ measurement	8*	For each variable of interest, give sources of data and details of methods of assessment (measurement). Describe comparability of assessment methods if there is more than one group	8, 9
Bias	9	Describe any efforts to address potential sources of bias	8, 10, 11
Study size	10	Explain how the study size was arrived at	10
Quantitative variables	11	Explain how quantitative variables were handled in the analyses. If applicable, describe which groupings were chosen and why	8-11
Statistical methods	12	(*a*) Describe all statistical methods, including those used to control for confounding	10, 11
		(*b*) Describe any methods used to examine subgroups and interactions	10, 11
		(*c*) Explain how missing data were addressed	10
		(*d*) If applicable, describe analytical methods taking account of sampling strategy	NA
		(*e*) Describe any sensitivity analyses	11
**Results**			

For peer review only

Participants	13*	(a) Report numbers of individuals at each stage of study—eg numbers potentially eligible, examined for eligibility, confirmed eligible, included in the study, completing follow-up, and analysed	10
		(b) Give reasons for non-participation at each stage	NA
		(c) Consider use of a flow diagram	
Descriptive data	14*	(a) Give characteristics of study participants (eg demographic, clinical, social) and information on exposures and potential confounders	13
		(b) Indicate number of participants with missing data for each variable of interest	13, 14
Outcome data	15*	Report numbers of outcome events or summary measures	14, 16-19
Main results	16	(*a*) Give unadjusted estimates and, if applicable, confounder-adjusted estimates and their precision (eg, 95% confidence interval). Make clear which confounders were adjusted for and why they were included	Table 2, Table 3, and Appendix Table 3
		(*b*) Report category boundaries when continuous variables were categorized	17, 18
		(*c*) If relevant, consider translating estimates of relative risk into absolute risk for a meaningful time period	NA
Other analyses	17	Report other analyses done—eg analyses of subgroups and interactions, and sensitivity analyses	15-19
**Discussion**			
Key results	18	Summarise key results with reference to study objectives	20
Limitations	19	Discuss limitations of the study, taking into account sources of potential bias or imprecision. Discuss both direction and magnitude of any potential bias	20, 21
Interpretation	20	Give a cautious overall interpretation of results considering objectives, limitations, multiplicity of analyses, results from similar studies, and other relevant evidence	21-24
Generalisability	21	Discuss the generalisability (external validity) of the study results	20
**Other information**			
Funding	22	Give the source of funding and the role of the funders for the present study and, if applicable, for the original study on which the present article is based	25

*Give information separately for cases and controls in case-control studies and, if applicable, for exposed and unexposed groups in cohort and cross-sectional studies.

Note: An Explanation and Elaboration article discusses each checklist item and gives methodological background and published examples of transparent reporting. The STROBE checklist is best used in conjunction with this article (freely available on the Web sites of PLoS Medicine at http://www.plosmedicine.org/, Annals of Internal Medicine at http://www.annals.org/, and Epidemiology at http://www.epidem.com/). Information on the STROBE Initiative is available at www.strobe-statement.org.

For peer review only

BMJ Open

Do sex differences in the prevalence of electrocardiographic abnormalities vary across ethnic groups living in the Netherlands? A cross-sectional analysis of the population-based HELIUS study

Journal:	*BMJ Open*
Manuscript ID	bmjopen-2020-039091.R1
Article Type:	Original research
Date Submitted by the Author:	25-Jun-2020
Complete List of Authors:	Bolijn, Renee; Amsterdam UMC, Public and Occupational Health<br>ter Haar, Cato; Amsterdam UMC, Cardiology; Leiden University Medical Center, Cardiology<br>Harskamp, Ralf; Amsterdam UMC, Department of General Practice<br>Tan, Hanno; Amsterdam UMC, Cardiology; ICIN-Netherlands Heart Institute<br>Kors, Jan; Erasmus Medical Center<br>Postema, Pieter; Amsterdam UMC, Clinical and Experimental Cardiology<br>Snijder, Marieke; Amsterdam UMC, Public and Occupational Health; Amsterdam UMC, Clinical Epidemiology, Biostatistics and Bioinformatics<br>Peters, Ron; Amsterdam UMC, Cardiology<br>Kunst, Anton; Amsterdam UMC, Public and Occupational Health<br>van Valkengoed, Irene; Amsterdam UMC, Public and Occupational Health
**Primary Subject Heading**:	Cardiovascular medicine
Secondary Subject Heading:	Public health, Epidemiology
Keywords:	Cardiac Epidemiology < CARDIOLOGY, Pacing & electrophysiology < CARDIOLOGY, EPIDEMIOLOGY, PUBLIC HEALTH

SCHOLARONE™
Manuscripts

I, the Submitting Author has the right to grant and does grant on behalf of all authors of the Work (as defined in the below author licence), an exclusive licence and/or a non-exclusive licence for contributions from authors who are: i) UK Crown employees; ii) where BMJ has agreed a CC-BY licence shall apply, and/or iii) in accordance with the terms applicable for US Federal Government officers or employees acting as part of their official duties; on a worldwide, perpetual, irrevocable, royalty-free basis to BMJ Publishing Group Ltd ("BMJ") its licensees and where the relevant Journal is co-owned by BMJ to the co-owners of the Journal, to publish the Work in this journal and any other BMJ products and to exploit all rights, as set out in our licence.

The Submitting Author accepts and understands that any supply made under these terms is made by BMJ to the Submitting Author unless you are acting as an employee on behalf of your employer or a postgraduate student of an affiliated institution which is paying any applicable article publishing charge ("APC") for Open Access articles. Where the Submitting Author wishes to make the Work available on an Open Access basis (and intends to pay the relevant APC), the terms of reuse of such Open Access shall be governed by a Creative Commons licence – details of these licences and which Creative Commons licence will apply to this Work are set out in our licence referred to above.

Other than as permitted in any relevant BMJ Author's Self Archiving Policies, I confirm this Work has not been accepted for publication elsewhere, is not being considered for publication elsewhere and does not duplicate material already published. I confirm all authors consent to publication of this Work and authorise the granting of this licence.

For peer review only

Do sex differences in the prevalence of electrocardiographic abnormalities vary across ethnic groups living in the Netherlands? A cross-sectional analysis of the population-based HELIUS study

Renee Bolijn[1], C. Cato ter Haar[2,3], Ralf E. Harskamp[4], Hanno L. Tan[2,5], Jan A. Kors[6], Pieter G. Postema[7], Marieke B. Snijder[1,8], Ron J.G. Peters[2], Anton E. Kunst[1], Irene G.M. van Valkengoed[1]

[1] *Amsterdam UMC, University of Amsterdam, Department of Public and Occupational Health, Amsterdam Public Health research institute, Meibergdreef 9, Amsterdam, The Netherlands.*

[2] *Amsterdam UMC, University of Amsterdam, Department of Cardiology, Amsterdam Cardiovascular Sciences, Meibergdreef 9, Amsterdam, The Netherlands.*

[3] *Leiden University Medical Center, Heart-Lung Center, Department of Cardiology, Leiden, The Netherlands.*

[4] *Amsterdam UMC, University of Amsterdam, Department of General Practice, Amsterdam Public Health research institute, Meibergdreef 9, Amsterdam, The Netherlands.*

[5] *Netherlands Heart Institute, Utrecht, The Netherlands.*

[6] *Department of Medical Informatics, Erasmus University Medical Center, Rotterdam, The Netherlands.*

[7] *Amsterdam UMC, University of Amsterdam, Department of Clinical and Experimental Cardiology, Amsterdam Cardiovascular Sciences, Meibergdreef 9, Amsterdam, The Netherlands.*

[8] *Amsterdam UMC, University of Amsterdam, Department of Clinical Epidemiology, Biostatistics and Bioinformatics, Amsterdam Public Health research institute, Meibergdreef 9, Amsterdam, The Netherlands*

For peer review only - http://bmjopen.bmj.com/site/about/guidelines.xhtml

Corresponding author: Renee Bolijn, Amsterdam UMC, University of Amsterdam, Department of Public and Occupational Health, Meibergdreef 9, 1105 AZ Amsterdam, The Netherlands. E-mail: r.bolijn@amsterdamumc.nl. Phone: +31 20 5664649

Word count: 3561

Keywords: ECG abnormalities, sex differences, ethnic minorities, cardiovascular disease prevention, HELIUS study

For peer review only

For peer review only - http://bmjopen.bmj.com/site/about/guidelines.xhtml

ABSTRACT

Objectives Major electrocardiographic (ECG) abnormalities have been associated with increased risk of cardiovascular disease (CVD) burden in asymptomatic populations. However, sex differences in occurrence of major ECG abnormalities have been poorly studied, particularly across ethnic groups. The objectives were to investigate 1) sex differences in the prevalence of major and, as a secondary outcome, minor ECG abnormalities, 2) whether patterns of sex differences varied across ethnic groups, by age, and 3) to what extent conventional cardiovascular risk factors contributed to observed sex differences.

Design Cross-sectional analysis of population-based study.

Setting Multi-ethnic, population-based Healthy Life in an Urban Setting (HELIUS) cohort, Amsterdam, the Netherlands.

Participants 8,089 men and 11,369 women of Dutch, South-Asian Surinamese, African Surinamese, Ghanaian, Turkish, and Moroccan origin aged 18-70 years without CVD.

Outcome measures Age-adjusted and multivariable logistic regression analyses were performed to study sex differences in prevalence of major and, as secondary outcome, minor ECG abnormalities in the overall population, across ethnic groups, and by age-groups (18-35, 36-50, and >50 years).

Results Major and minor ECG abnormalities were less prevalent in women than men (4.6% versus 6.6%, and 23.8% versus 39.8%, respectively). After adjustment for conventional risk factors, sex differences in major abnormalities were smaller in ethnic minority groups (odds ratios [OR] ranged from 0.61 in Moroccans to 1.32 in South-Asian Surinamese) than in the Dutch (OR 0.49; 95% confidence interval [CI] 0.36-0.65). Only in South-Asian Surinamese, women did not have a lower odds than men (OR 1.32; 95% CI 0.96-1.84). The pattern of smaller sex differences in ethnic minority groups was more pronounced in older than in younger age-groups.

For peer review only - http://bmjopen.bmj.com/site/about/guidelines.xhtml

Conclusions The prevalence of major ECG abnormalities was lower in women than men. However, sex differences were less apparent in ethnic minority groups. Conventional risk factors did not contribute substantially to observed sex differences.

For peer review only

Strengths and limitations of this study

- Participants were sampled from the municipality registry and reflect a general population sample of adults of the major ethnic groups living in Amsterdam.
- Large sample sizes permit the study of sex differences within each ethnic group, including across age strata.
- Single electrocardiographic (ECG) measurements of 10 seconds without additional imaging techniques (e.g., echocardiography) may be suboptimal for the measurement of ECG abnormalities.
- The classification of 'major' and 'minor' ECG abnormalities may depend on criteria used, which may affect reported prevalence estimates.

For peer review only

For peer review only - http://bmjopen.bmj.com/site/about/guidelines.xhtml

INTRODUCTION

The resting electrocardiogram (ECG) is an essential diagnostic instrument in patients with symptoms suggestive of cardiovascular disease (CVD).[1] Previous studies show that the occurrence of major ECG abnormalities is also associated with increased risk of CVD morbidity[2,3] and mortality[3,4] in asymptomatic populations. However, differences in the occurrence of major ECG abnormalities in men and women have been poorly studied. Insights in these sex differences may help to identify subpopulations with a future CVD burden and thus aid targeted (preventive) therapy.

Although studies have described the prevalence of major ECG abnormalities in men and women from diverse general populations,[5-11] only three studied sex differences in general populations specifically.[5-7] Whether the occurrence of ECG abnormalities differs by sex, independently of cardiovascular risk factors, is a topic of ongoing debate. Two studies suggested that the composite of major ECG abnormalities (e.g., atrial fibrillation, Q-wave or T-wave abnormalities) is more prevalent among men than women,[5,6] while another observed no sex differences.[7]

Differences in the occurrence of ECG abnormalities have been observed between ethnic groups living in similar contexts.[6,8,12,13] However, in Europe, the prevalence of major ECG abnormalities among ethnic minority populations at high risk for CVD, such as men and women of South-Asian origin,[14] is unknown. Additionally, it is unknown to what extent major ECG abnormalities vary between men and women across ethnic groups.

The prevalence of ECG abnormalities tends to increase with increasing age.[5] As larger sex differences in occurrence of CVD have been found in younger age-groups compared to older age-groups,[15] sex differences in prevalence of ECG abnormalities may also vary by age.

For peer review only

In this study, we investigated sex differences in the prevalence of major and, as a secondary outcome, minor ECG abnormalities, in a 18-70 year-old multi-ethnic population living in Amsterdam, the Netherlands. We assessed whether patterns of sex differences varied across ethnic groups, overall and by age, and to what extent conventional cardiovascular risk factors contributed to observed sex differences, overall and within subgroups.

For peer review only

For peer review only - http://bmjopen.bmj.com/site/about/guidelines.xhtml

METHODS

We used baseline data from the HEalthy LIfe in an Urban Setting (HELIUS) study, a multi-ethnic cohort study conducted in Amsterdam, the Netherlands. The HELIUS study has been described in detail elsewhere.[16,17] Briefly, baseline data collection took place between 2011 and 2015 and included participants of Dutch, South-Asian Surinamese, African Surinamese, Ghanaian, Moroccan, and Turkish origin aged 18-70 years living in Amsterdam. Potential participants were sampled with a simple random sampling method from the municipality registry, after stratification by ethnicity as defined by registered country of birth.[18] Data were obtained by questionnaire and physical examinations (including biological samples). The HELIUS study has been approved by the AMC Ethical Review Board. All participants provided written informed consent.

ECG measurements

Standard 12-lead ECGs were recorded in supine position with a GE MAC5500 electrocardiograph at 500 samples/sec and analysed using the Modular ECG Analysis System (MEANS).[19] The measurement of ECG abnormalities has been described in detail elsewhere.[20] Briefly, ECG abnormalities were assessed by combining ECG diagnoses of the MEANS programs with Minnesota coding, Marquette 12SL ECG analysis software, and a cardiologist's interpretation. In case of discrepancies, ECGs were double checked. We classified ECG abnormalities into major and minor ECG abnormalities, based on previous research[7] and consensus discussion among experts (Appendix Table 1). This classification was completed prior to data analysis.

Ethnicity

Ethnicity was defined by the individual's country of birth combined with the parental countries of birth.[18] Surinamese participants were further classified according to self-reported ethnic origin into "African", "South-Asian", "Javanese", or "other".

For peer review only - http://bmjopen.bmj.com/site/about/guidelines.xhtml

Covariables

Family history of CVD was defined by a self-reported CVD diagnosis among first degree family members aged <60 years. Smoking was classified as current, past, or never smoker. For current smokers, the number of pack-years of smoking was calculated by multiplying the number of packs (containing 20 cigarettes or equivalent rates for cigars and pipe tobacco) smoked a day by the number of years smoked. Physical activity was defined as achieving ≥30 minutes of moderate- or high-intensity activity per day on ≥5 days per week.[21] Alcohol consumption (on average in the last 12 months) was classified as: none or low (men: 0-4; women: 0-2 beverages/week), moderate (men: 5-14; women: 3-7 beverages/week), and high (men: >14; women: >7 beverages/week).

Body mass index (BMI) was calculated in duplicate as weight (kg) divided by height squared (m^2). Blood pressure (BP) was measured in duplicate using a validated automated digital BP device (WatchBP Home; Microlife AG) in a seated position after ≥5 minutes of rest. Hypertension was defined as systolic BP ≥140 mm Hg, diastolic BP ≥90 mm Hg, use of antihypertensive medication treatment, and/or self-reported hypertension.

Fasting blood samples were drawn to determine creatinine, lipid, and glucose concentrations (details on these measurements have been described elsewhere).[22] Chronic kidney disease (CKD) risk was categorized according to the risk of progression of kidney disease based on estimated Glomerular Filtration Rate and albuminuria levels:[23] (1) low, (2) moderately increased, (3) high and very high risk. Hypercholesterolemia was defined as total cholesterol ≥5.0 mmol/l, high-density lipoprotein cholesterol <1.0 mmol/l (men) or <1.2 mmol/l (women), low-density lipoprotein cholesterol ≥3.0 mmol/l (Friedewald formula[24]), triglycerides ≥1.7 mmol/l, use of lipid-lowering medication, and/or self-reported hypercholesterolemia. Participants were considered to have diabetes in case of a fasting

For peer review only - http://bmjopen.bmj.com/site/about/guidelines.xhtml

glucose ≥7.0 mmol/l, use of glucose-lowering medication and/or if they reported to be diagnosed with diabetes by a doctor.

Study population

Baseline data were available for 22,165 participants. We excluded those of Javanese Surinamese (n=233), unknown Surinamese (n=267) origin, and with another/unknown ethnic origin (n=48). Next, we excluded participants with a history of CVD (n=1,610; based on self-reported prior myocardial infarction, cerebrovascular accident (CVA), angioplasty or bypass surgery (on heart or legs), use of antiplatelet drugs (Anatomical Therapeutic Chemical [ATC] code B01AC), use of oral anticoagulants (ATC codes B01AA, B01AE, B01AF), use of antiarrhythmic agents (ATC-codes C01A, C01B, C07AA07, C08D), or paced rhythms). Finally, we excluded participants with missing ECG data (n=337) or with missing data on ≥1 covariables (n=212), resulting in a study population of 19,458 participants (Appendix Figure 1).

Statistical analyses

Baseline characteristics were expressed as means (standard deviations [SD]) or frequencies (percentages) by sex in the total population and per ethnic group. The age-adjusted prevalence of any major ECG abnormality, any minor ECG abnormality, and a selection of common ECG abnormalities (i.e., major ECG abnormalities with a prevalence of ≥1% and the top 5 most prevalent minor ECG abnormalities) was calculated by sex, in the total population and by ethnicity, using the study population as the standard. For reference, the overall prevalence of less common ECG abnormalities is also provided, but only by sex in the total population. The prevalence of any major ECG abnormalities was also calculated by age-groups (i.e., 18-35, 36-50, and >50 years based on tertiles of the age distribution in the total population) for all ethnic groups.

For peer review only - http://bmjopen.bmj.com/site/about/guidelines.xhtml

We performed binary logistic regression analyses with hierarchal models to examine sex differences in prevalence of 1) any major ECG abnormalities and 2) any minor ECG abnormalities, adjusted for age and ethnicity (model 1), and additionally for hypertension, hypercholesterolemia, diabetes, and smoking status (model 2) to determine to what extent conventional cardiovascular risk factors contributed to observed differences. We also examined the additional contribution of other well-known cardiovascular risk factors, i.e., family history of CVD and CKD risk (model 3) and BMI, alcohol consumption, and physical activity (model 4). To study whether the sex differences varied between ethnic groups (i.e., effect modification), a statistical interaction term for sex and ethnicity on a multiplicative scale was added. Then, the main analyses (model 2 with interaction term) for major ECG abnormalities were repeated stratified by age-groups (18-35, 36-50, and >50 years) to examine the consistency of sex differences across ethnic groups among age-groups. All statistical analyses were performed in R studio version 1.1.453.[25] p-values <0.05 were regarded as statistically significant.

Sensitivity analyses

We repeated the main analyses excluding obese participants (BMI >30), since obesity may influence the accuracy of ECG measurements.[26] Furthermore, use of psychotropic medication may induce alterations of the ECG resulting in ECG abnormalities (e.g., QT prolongation).[27] Therefore, we repeated the analyses excluding participants with current use of psychotropic medication. Finally, we repeated the analyses using number of pack-years of smoking instead of smoking status, to examine whether the scale of the variables (numeric versus categorical) altered the results.

Patient and public involvement

There was no specific patient or public involvement in the development of the research questions, outcome measures, study design, and recruitment/conduct of the present study. However, for the core HELIUS study, several supportive measures were taken to enhance the enrolment of ethnic

For peer review only - http://bmjopen.bmj.com/site/about/guidelines.xhtml

minority groups. For example, ethnic-specific communication strategies were used, such as working with faith communities (churches and mosques) and endorsement from local key figures. Understandability of and time to complete the questionnaire were also enquired among participants. In addition, the present study is part of a larger project on sex and gender differences in CVD risk. As part of this project, interviews and a short survey on research priority setting according to CVD patients and persons at increased CVD risk were conducted. The present study aligns with our findings from these interviews and survey that more research on sex and gender differences in CVD was perceived as relevant by the target group.

For peer review only

For peer review only - http://bmjopen.bmj.com/site/about/guidelines.xhtml

RESULTS

Mean age was around 43 years (SD 13) in women and 44 years (SD 13) in men (Table 1). More than 20% of both men and women had a family history of CVD. Women were less often current smokers and had fewer mean pack-years of smoking compared to men, while the prevalence of high alcohol consumption was similar among men and women. Women had a higher mean BMI and were less physically active. Hypertension, hypercholesterolemia, and diabetes were less prevalent among women than men, while high CKD risk was equally prevalent among men and women. Women more often used psychotropic medication than men. These patterns in baseline characteristics differed across ethnic groups (Appendix Table 2).

Table 1. Baseline characteristics of 19,458 men and women with ECG measurements

	**Men (n=8,089)**	**Women (n=11,369)**
Age (years)	43.8 (13.0)	43.1 (13.0)
Ethnicity		
Dutch	1,873 (23.2)	2,293 (20.2)
South-Asian Surinamese	1,125 (13.9)	1,464 (12.9)
African Surinamese	1,411 (17.4)	2,266 (19.9)
Ghanaian	822 (10.2)	1,321 (11.6)
Turkish	1,451 (17.9)	1,769 (15.6)
Moroccan	1,407 (17.4)	2,256 (19.8)
Family history of CVD (missing: n=217)	1,637 (20.4)	2,611 (23.3)
Smoker		
Current	2,539 (31.4)	2,032 (17.9)
Past	2,021 (25.0)	1,753 (15.4)
Never	3,529 (43.6)	7,584 (66.7)
Pack-years of smoking (missing: n=191)	5.4 (16.2)	1.8 (7.3)

For peer review only - http://bmjopen.bmj.com/site/about/guidelines.xhtml

	Men (n=8,089)	Women (n=11,369)
Achieving physical activity norm (missing: n=27)	5,020 (62.2)	5,963 (52.5)
Alcohol consumption (missing: n=115)		
None or low	5,981 (74.4)	8,985 (79.5)
Moderate	1,526 (19.0)	1,549 (13.7)
High	528 (6.6)	774 (6.8)
BMI (kg/m^2; missing: n=15)	26.3 (4.2)	27.5 (5.8)
CKD risk (missing: n=63)		
Low	7,684 (95.4)	10,689 (94.3)
Moderate	304 (3.8)	555 (4.9)
High	68 (0.8)	95 (0.8)
Hypertension	3,026 (37.4)	3,594 (31.6)
Hypercholesterolemia	5,752 (71.1)	7,147 (62.9)
Diabetes	829 (10.2)	952 (8.4)
Use of psychotropic medication (missing: n=4)[a]	397 (4.9)	679 (6.0)

Data are presented as means (standard deviations) or frequencies (percentages).

BMI, body mass index; CKD, chronic kidney disease; CVD, cardiovascular disease.

[a] Anatomical Therapeutic Chemical (ATC) codes: N03AE, N03AF, N03AG, N03AN, N05A, N05BA, N05C, N06A, N06BA, N07B, and R06AD02

For peer review only

Overall, the age-adjusted prevalence of major ECG abnormalities was lower among women (4.6%) compared to men (6.6%; Table 2). In most ethnic groups, women had a lower age-adjusted prevalence (range: 2.9%-6.1%) compared to men (range: 4.7%-7.9%), except in the South-Asian Surinamese (7.2% versus 6.0% respectively). Conventional cardiovascular risk factors and other well-known risk factors did not contribute substantially to the observed sex differences in major ECG abnormalities in the total population and within ethnic groups. For instance, the odds ratio (OR) of having a major ECG abnormality changed from 0.69 (95% confidence interval [CI] 0.61-0.78) to 0.71 (95% CI 0.62-0.81)

For peer review only - http://bmjopen.bmj.com/site/about/guidelines.xhtml

among women versus men after adjustment for hypertension, hypercholesterolemia, diabetes, and smoking status, and to 0.67 (95% CI 0.58-0.76) after adjustment for family history of CVD, CKD risk, BMI, alcohol consumption, and physical activity.

There was a general pattern of smaller sex differences in occurrence of major ECG abnormalities in the ethnic minority groups compared to the Dutch (Table 2). The adjusted OR for women versus men varied from 0.49 (95% CI 0.36-0.65) in the Dutch to 0.73 (95% CI 0.53-1.01) in Turkish. Only in the South-Asian Surinamese group, women did not have a lower odds than men (adjusted OR 1.32; 95% CI 0.96-1.84).

For peer review only

For peer review only - http://bmjopen.bmj.com/site/about/guidelines.xhtml

Table 2. Number of cases and age-adjusted prevalence of any major ECG abnormality by sex in the total population and by ethnic group, and the odds of major ECG abnormalities in women compared to men, overall and with an interaction term for sex and ethnicity

	Men	Women	Model 1				Model 2			
	(n of cases, %[a])	(n of cases, %[a])	OR (95% CI)	*p*-value	Ratio of ORs (95% CI)*	*p*-value	OR (95% CI)	*p*-value	Ratio of ORs (95% CI)*	*p*-value
Overall	540 (6.6)	518 (4.6)	0.69 (0.61-0.78)[b]	*<0.001*	NA	NA	0.71 (0.62-0.81)[b]	*<0.001*	NA	NA
Dutch	137 (7.3)	79 (3.6)	0.46 (0.34-0.61)	*<0.001*	Reference	NA	0.49 (0.36-0.65)	*<0.001*	Reference	NA
SA Surinamese	63 (6.0)	110 (7.2)	1.24 (0.90-1.72)	0.19	2.69 (1.75-4.17)	*<0.001*	1.32 (0.96-1.84)	0.09	2.72 (1.76-4.21)	*<0.001*
African Surinamese	107 (7.6)	118 (5.3)	0.68 (0.51-0.89)	*<0.01*	1.47 (0.99-2.18)	0.06	0.68 (0.52-0.90)	*<0.01*	1.40 (0.94-2.09)	0.10
Ghanaian	72 (7.9)	71 (6.1)	0.70 (0.49-0.98)	*0.04*	1.51 (0.97-2.37)	0.07	0.71 (0.51-1.01)	0.055	1.47 (0.94-2.30)	0.09
Turkish	89 (6.1)	77 (4.4)	0.71 (0.52-0.98)	*0.04*	1.55 (1.01-2.37)	*0.045*	0.73 (0.53-1.01)	0.058	1.51 (0.98-2.32)	0.06
Moroccan	72 (4.7)	63 (2.9)	0.59 (0.42-0.84)	*<0.01*	1.29 (0.82-2.02)	0.27	0.61 (0.43-0.87)	*<0.01*	1.26 (0.80-1.99)	0.33

CI, confidence interval; NA, not applicable; OR, odds ratio; SA, South-Asian.

Significant *p*-values (p<0.05) are printed in italic.

Model 1: adjusted for age; model 2: adjusted for age, hypertension, hypercholesterolemia, diabetes, and smoking status

[a] Age-adjusted prevalence.

[b] Additionally adjusted for ethnicity.

* Measure of effect modification on multiplicative scale (statistical interaction term).

For peer review only

For peer review only - http://bmjopen.bmj.com/site/about/guidelines.xhtml

In the total population, the most frequently observed major ECG abnormalities were T-wave abnormalities (1.2%), microvoltages (1.2%), and (ECG suggestive of) cardiomyopathy (1.1%) among women and T-wave abnormalities (1.6%), right bundle branch block (RBBB; 1.4%), and cardiomyopathy (1.1%) among men. Among South-Asian Surinamese women, the only group of women with no lower odds than men, microvoltages (2.9%), cardiomyopathy (1.9%), and T-wave abnormalities (1.5%) were the most prevalent major ECG abnormalities. T-wave abnormalities (1.9%), cardiomyopathy (1.2%), and RBBB (1.1%) were the most prevalent major ECG abnormalities among South-Asian Surinamese men.

As expected, the prevalence of major ECG abnormalities was higher in older than younger age-groups in both men and women (Figure 1). The general pattern of smaller sex differences in the ethnic minority groups compared to the Dutch differed across the age strata (Table 3). In the older age groups, the adjusted sex difference in the odds of having a major ECG abnormality appeared less pronounced in ethnic minorities compared to the Dutch, whereas this sex difference appeared more similar across ethnic group in the youngest age group. Whereas women in all ethnic groups had a lower odds compared to men across all age strata, this was only the case in the youngest age-group of South-Asian Surinamese women versus men.

Table 3. The odds of major ECG abnormalities in women compared to men by age-groups, in the total population and with an interaction term for sex and ethnicity

**Aged 18-35 years (n=5870)[a]**	**OR (95% CI)**	***p*-value**	**Ratio of ORs (95% CI)***	***p*-value**
Overall[b]	0.38 (0.27-0.54)	*<0.001*	NA	NA
Dutch	0.30 (0.14-0.60)	*<0.01*	Reference	NA
South-Asian Surinamese	0.48 (0.18-1.18)	0.12	1.58 (0.47-5.11)	0.45
African Surinamese	0.38 (0.12-1.05)	0.07	1.25 (0.34-4.44)	0.73

For peer review only - http://bmjopen.bmj.com/site/about/guidelines.xhtml

Ghanaian	0.48 (0.13-1.77)	0.26	1.60 (0.37-7.07)	0.52
Turkish	0.55 (0.28-1.09)	0.09	1.84 (0.69-5.01)	0.23
Moroccan	0.23 (0.09-0.55)	*<0.01*	0.77 (0.23-2.42)	0.65
**Aged 36-50 years (n=7099)[a]**	**OR (95% CI)**	***p*-value**	**Ratio of ORs (95% CI)***	***p*-value**
Overall[b]	0.89 (0.70-1.12)	0.32	NA	NA
Dutch	0.51 (0.28-0.90)	*0.02*	Reference	NA
South-Asian Surinamese	2.61 (1.39-5.20)	*<0.01*	5.13 (2.19-12.56)	*<0.001*
African Surinamese	0.49 (0.29-0.85)	*0.01*	0.97 (0.44-2.16)	0.94
Ghanaian	0.91 (0.50-1.71)	0.76	1.79 (0.78-4.22)	0.18
Turkish	1.04 (0.64-1.69)	0.87	2.04 (0.97-4.38)	0.06
Moroccan	0.92 (0.51-1.67)	0.79	1.81 (0.80-4.17)	0.16
**Aged >50 years (n=6489)[a]**	**OR (95% CI)**	***p*-value**	**Ratio of ORs (95% CI)***	***p*-value**
Overall[b]	0.74 (0.62-0.89)	*<0.01*	NA	NA
Dutch	0.54 (0.37-0.78)	*<0.01*	Reference	NA
South-Asian Surinamese	1.22 (0.79-1.91)	0.37	2.27 (1.28-4.06)	*<0.01*
African Surinamese	0.84 (0.59-1.18)	0.31	1.55 (0.94-2.59)	0.09
Ghanaian	0.74 (0.46-1.17)	0.20	1.37 (0.75-2.48)	0.30
Turkish	0.55 (0.31-0.96)	*0.04*	1.02 (0.51-2.00)	0.96
Moroccan	0.68 (0.40-1.16)	0.16	1.26 (0.66-2.42)	0.49

CI, confidence interval; NA, not applicable; OR, odds ratio.

[a] Model adjustment: age, hypertension, hypercholesterolemia, diabetes, and smoking status.

[b] These models were also adjusted for ethnicity.

* Measure of effect modification on multiplicative scale (statistical interaction term).

Women had a lower prevalence of minor ECG abnormalities (range: 16.2%-35.6%) compared to men (range: 28.5%-55.7%; Appendix Table 3). Sex differences in minor ECG abnormalities were similar across ethnic groups, and were not influenced by conventional risk factors.

For peer review only

The prevalence of most common ECG abnormalities was also lower in women than men (Appendix Table 4a). Only mildly prolonged QTc interval was more prevalent in women than in men. Patterns were similar across ethnic groups. The prevalence of most less common ECG abnormalities was also lower in women than women, except for microvoltages, severely prolonged QTc (Bazett) interval, and left bundle branch block (LBBB), atrial rhythm, and sinus tachycardia (Appendix Table 4b).

Sensitivity analyses did not alter our interpretation of findings (data not shown).

For peer review only

DISCUSSION

In our study, women have an overall lower age-adjusted prevalence of major ECG abnormalities than men. Sex differences in the prevalence of major ECG abnormalities are smaller in the ethnic minority groups than in the Dutch, particularly in older age-groups. Differences in conventional cardiovascular risk factors and other well-known risk factors do not contribute substantially to these sex differences.

Our study has limitations. First, the results may be affected by selection bias due to non-response (response rate: 28%). Non-response analyses showed that women were more likely to participate than men, Turks and Moroccans were less likely to participate compared to other ethnic groups, and participants were slightly older than non-participants.[17] However, we were able to include large numbers of both men and women, each ethnic group, and age-group, indicating sufficient representation of all subgroups. This is relevant because previous work has shown that relative differences in CVD risk factors between ethnic groups are similar to other European countries,[28] suggesting that our results are generalizable to other European countries. Second, the definition of prior CVD was not comprehensive, as data on self-reported prior CVD other than myocardial infarction and CVA were lacking. However, we also excluded participants with a prior angioplasty or bypass surgery (on heart or legs), or paced rhythms, and those participants using antiplatelet drugs, oral anticoagulants, or antiarrhythmic agents, and verified that our results were consistent in analyses restricted to those with a favourable cardiovascular risk profile (post-hoc analysis in participants without hypertension, hypercholesterolemia, diabetes, and those not smoking; data not shown). Therefore, it is unlikely that our results were substantially affected by misclassification. Third, single ECG measurements may have been suboptimal for the measurement of ECG abnormalities, potentially affecting the prevalence estimates. Some common expressions of CVD might not always be detectable by a single ECG measurement of 10 seconds, such as paroxysmal atrial fibrillation, and some ECG abnormalities need additional diagnostic measurements. However, 24-hour ECG monitoring with

For peer review only - http://bmjopen.bmj.com/site/about/guidelines.xhtml

portable ECG devices and additional imaging techniques (e.g., echocardiography) are often not feasible in population-based studies. Finally, the classification of ‘major’ and ‘minor’ ECG abnormalities depends on criteria used, and may be variable given the complexity of detailed ECG interpretation. For instance, the level of severity of some abnormalities may depend on the full clinical assessment or on combinations of abnormalities (e.g., RBBB with left axis deviation). We also did not distinguish between men and women, ethnic groups, and age-groups in the assessment of the classification of major and minor abnormalities. If future research would reveal that the implication of abnormalities is different for any of these groups, this may influence the magnitude of the observed differences in our study.

Similar to previous studies reporting on the prevalence of composite major ECG abnormalities stratified by sex,[5-8,10,11] we observed an overall lower prevalence of major ECG abnormalities in women compared to men in most ethnic groups. Prevalence estimates in both men and women were within the range reported in most previous studies (range: 3.0%-13.2%),[5,6,8,10] except two studies with higher estimates.[7,11] T-wave abnormalities were the most prevalent major ECG abnormalities in both men and women in our study and most previous studies.[5-7,10,11] A much larger heterogeneity has been reported in previous studies in prevalence of minor ECG abnormalities, ranging from 4.5% to 31.6% in women[5,6,8,9,11] and from 7.3% to 45.7% in men.[5,6,8,9,11] Our prevalence was higher compared to most studies, most likely due to differences in the classification of major and minor ECG abnormalities.

The observed sex differences in major ECG abnormalities are in line with known differences in cardiovascular pathophysiology and epidemiology of CVD between men and women.[29,30] For instance, men tend to develop coronary artery disease (CAD) earlier than women, resulting in a higher incidence of CAD in men compared to women, in particular at a younger age.[29,30] This age-effect is

For peer review only - http://bmjopen.bmj.com/site/about/guidelines.xhtml

consistent with our observations across ethnic groups of larger sex differences in prevalence of major ECG abnormalities in the youngest age-group compared to the older age-groups.

Differential patterning of cardiovascular risk factors did not explain the observed sex differences in prevalence of major ECH abnormalities overall and across ethnic groups. This finding is consistent with two previous studies on sex differences in ECG abnormalities[5,6] but not with another study.[7] Other explanations for the relative cardiovascular advantage of women compared to men at a younger age are still unclear, but may relate to sex hormones, with a prominent role for the protective effects of estrogen in the development of CVD among premenopausal women.[29] Our findings of larger sex differences in prevalence of major ECG abnormalities in the youngest age-group compared to the older age-groups support this hypothesis.

We observed that only South-Asian Surinamese women did not have a lower odds of having a major ECG abnormality compared to South-Asian Surinamese men, which was mainly due to the higher prevalence of major ECG abnormalities among South-Asian Surinamese women compared to other women. South-Asian populations living in Europe are already considered a high-risk population for CVD[14] and our findings may suggest that South-Asian Surinamese women specifically are a target group for CVD prevention strategies. Although women had a consistently lower odds of having a major ECG abnormality than men in all other ethnic groups (except South-Asian Surinamese), Dutch women had a larger cardiovascular advantage than the other women. These findings are in line with a previous study from the USA showing a larger gap between men and women of the white majority population compared to black men and women in CAD mortality.[31] In contrast, a Dutch study on sex disparities in myocardial infarction incidence observed a smaller sex difference in the Dutch majority population compared to minority populations originating from Morocco, South-Asia, and Turkey.[15] Explanations

For peer review only - http://bmjopen.bmj.com/site/about/guidelines.xhtml
For peer review only

for the discrepancy between this and our study are unclear, but may relate to differences in study populations and exclusion criteria.

Differential patterning of cardiovascular risk factors did not explain the smaller cardiovascular advantage among minority women compared to Dutch women, suggesting that other factors may be relevant. Psychosocial factors (e.g., discrimination), for instance, may be important risk factors for major ECG abnormalities in some groups of participants, potentially through stress and lifestyle-related factors. For instance, an American study found that current and chronic stress were associated with subclinical atherosclerosis in South-Asian women but not in South-Asian men.[32] Further research needs to confirm whether these psychosocial factors may also explain ethnic-specific variation in sex differences in occurrence of major ECG abnormalities.

The observed sex differences in occurrence of major ECG abnormalities, overall and within subgroups, may also reflect that ECG reference values do not differentiate between men and women (except QTc duration), ethnic groups, or age-groups. Normal values for ECGs may differ for women[33] and non-white groups[34] compared to white men, in whom the ECG reference criteria were developed. This is problematic since subgroups with pathological ECGs and potentially related cardiovascular risk might have been missed, or have a false positive diagnosis. For example, some studies suggest that current ECG criteria for microvoltages may be less valid for women[35] and Asian populations,[36] which may have resulted in an overestimation of the occurrence of microvoltages among South-Asian Surinamese women in our study.

In conclusion, we observed sex differences in ECG abnormalities and identified subpopulations with a relatively high prevalence, e.g., Dutch men, and men and women of South-Asian and African origin. Given the association of major ECG abnormalities with CVD morbidity and mortality,[2-4] these groups

For peer review only - http://bmjopen.bmj.com/site/about/guidelines.xhtml

may particularly benefit from prevention strategies to reduce the future burden of CVD. Moreover, the observed differences occurred irrespective of conventional risk factors, suggesting that opportunities to reduce the burden of CVD might be missed if prevention strategies are solely targeted at those with conventional risk factors. Previous studies have suggested that ECG measures may be, in addition to established cardiovascular risk factors, useful for the prediction of future CVD in intermediate and high-risk groups.[37,38] However, evidence is still limited, potential harms of screening are unknown, and ECG reference values are not sex-, ethnic-, and age-specific. Hence, screening for CVD with ECG is currently not recommended.[39] In future research, ECG reference values should be validated in ethnically diverse populations of men and women of different age-groups in order to further investigate the occurrence of ECG abnormalities and the potentially added value of an ECG to cardiovascular risk classification.

For peer review only

For peer review only - http://bmjopen.bmj.com/site/about/guidelines.xhtml

ACKNOWLEDGEMENTS

We are most grateful to the participants of the HELIUS study and the management team, research nurses, interviewers, research assistants and other staff who have taken part in gathering the data of this study.

COMPETING INTERESTS

None declared.

FUNDING

The HELIUS study is conducted by the Academic Medical Center Amsterdam and the Public Health Service of Amsterdam. Both organizations provided core support for HELIUS. The HELIUS study is also funded by the Dutch Heart Foundation, the Netherlands Organization for Health Research and Development (ZonMw), European Union (FP-7), and the European Fund for the Integration of non-EU immigrants (EIF). This work was additionally supported by a grant from the ZonMw Gender and Health Program (grant number 849200008). HLT has received funding from the European Union's Horizon 2020 research and innovation programme under acronym ESCAPE-NET (grant number 733381).

DATA AVAILABILITY STATEMENT

Data are available upon reasonable request.

For peer review only - http://bmjopen.bmj.com/site/about/guidelines.xhtml

For peer review only

AUTHORS' CONTRIBUTIONS

RB and IGMvV contributed to the conception and design of the work; CCtH, REH, PGP, and AEK contributed to the design. MBS and RJGP contributed to the acquisition of the data; all authors contributed to the analysis and interpretation of the results. RB drafted the manuscript. CCtH, REH, HLT, JAK, PGP, MBS, RJGP, AEK, and IGMvV critically revised the manuscript. All authors gave final approval and agree to be accountable for all aspects of work ensuring integrity and accuracy.

For peer review only

For peer review only - http://bmjopen.bmj.com/site/about/guidelines.xhtml

REFERENCES

[1] Fye WB. A history of the origin, evolution, and impact of electrocardiography. *Am J Cardiol* 1994;73:937-949.

[2] Denes P, Larson JC, Lloyd-Jones DM, et al. Major and minor ECG abnormalities in asymptomatic women and risk of cardiovascular events and mortality. *JAMA* 2007;297:978-985.

[3] Machado DB, Crow RS, Boland LL, et al. Electrocardiographic findings and incident coronary heart disease among participants in the Atherosclerosis Risk in Communities (ARIC) study. *Am J Cardiol* 2006;97:1176-1181.

[4] De Bacquer D, De Backer G, Kornitzer M, et al. Prognostic value of ECG findings for total, cardiovascular disease, and coronary heart disease death in men and women. *Heart* 1998;80:570-577.

[5] De Bacquer D, De Backer G and Kornitzer M. Prevalences of ECG findings in large population based samples of men and women. *Heart* 2000;84:625-633.

[6] Vitelli LL, Crow RS, Shahar E, et al. Electrocardiographic findings in a healthy biracial population. *Am J Cardiol* 1998;81:453-459.

[7] Prineas RJ, Le A, Soliman EZ, et al. United States national prevalence of electrocardiographic abnormalities in black and white middle-age (45-to 64-year) and older (≥65-year) adults (from the Reasons for Geographic and Racial Differences in Stroke Study). *Am J Cardiol* 2012;109:1223-1228.

[8] Walsh III JA, Prineas R, Daviglus ML, et al. Prevalence of electrocardiographic abnormalities in a middle-aged, biracial population: Coronary Artery Risk Development in Young Adults study. *J Electrocardiol* 2010;43:385.e1-385.e9.

[9] Liao YL, Liu KA, Dyer A, et al. Major and minor electrocardiographic abnormalities and risk of death from coronary heart disease, cardiovascular diseases and all causes in men and women. *J Am Coll Cardiol* 1988;12:1494-1500.

For peer review only - http://bmjopen.bmj.com/site/about/guidelines.xhtml

[10] Pinto-Filho MM, Brant LC, Foppa M, et al. Major electrocardiographic abnormalities according to the Minnesota Coding System among Brazilian adults (from the ELSA-Brasil Cohort Study). *Am J Cardiol* 2017;119:2081-2087.

[11] Wu C-C, Yeh W-T, Crow RS, et al. Comparison of electrocardiographic findings and associated risk factors between Taiwan Chinese and US White adults. *Int J Cardiol* 2008;128:224-231.

[12] Perez MV, Yaw TS, Myers J, et al. Prognostic value of the computerized ECG in Hispanics. *Clin Cardiol* 2007;30:189-194.

[13] Santhanakrishnan R, Wang N, Larson MG, et al. Racial differences in electrocardiographic characteristics and prognostic significance in Whites versus Asians. *J Am Heart Assoc* 2016;5:e002956.

[14] Cainzos-Achirica M, Fedeli U, Sattar N, et al. Epidemiology, risk factors, and opportunities for prevention of cardiovascular disease in individuals of South Asian ethnicity living in Europe. *Atherosclerosis* 2019;286:105-113.

[15] van Oeffelen AA, Vaartjes I, Stronks K, et al. Sex disparities in acute myocardial infarction incidence: Do ethnic minority groups differ from the majority population? *Eur J Prev Cardiol* 2015;22:180-188.

[16] Stronks K, Snijder MB, Peters RJ, et al. Unravelling the impact of ethnicity on health in Europe: the HELIUS study. *BMC Public Health* 2013;13:402.

[17] Snijder MB, Galenkamp H, Prins M, et al. Cohort profile: the Healthy Life in an Urban Setting (HELIUS) study in Amsterdam, the Netherlands. *BMJ Open* 2017;7:e017873.

[18] Stronks K, Kulu-Glasgow I and Agyemang C. The utility of 'country of birth' for the classification of ethnic groups in health research: the Dutch experience. *Ethn Health* 2009;14:255-269.

[19] van Bemmel J, Kors J and Van Herpen G. Methodology of the modular ECG analysis system MEANS. *Methods Inf Med* 1990;29:346-353.

For peer review only - http://bmjopen.bmj.com/site/about/guidelines.xhtml

[20] ter Haar C, Kors J, Peters R, et al. The prevalence of electrocardiograms exceeding thresholds for ST-elevation myocardial infarction in apparently healthy individuals: the role of ethnicity. *J Am Heart Assoc* 2020:Accepted for publication.

[21] Wendel-Vos GW, Schuit AJ, Saris WH, et al. Reproducibility and relative validity of the short questionnaire to assess health-enhancing physical activity. *J Clin Epidemiol* 2003;56:1163-1169.

[22] Agyemang C, Snijder MB, Adjei DN, et al. Ethnic disparities in CKD in the Netherlands: the HEalthy LIfe in an Urban Setting (HELIUS) study. *Am J Kidney Dis* 2016;67:391-399.

[23] Levin A, Stevens PE, Bilous RW, et al. Kidney Disease: Improving Global Outcomes (KDIGO) CKD Work Group. KDIGO 2012 clinical practice guideline for the evaluation and management of chronic kidney disease. *Kidney Int Suppl* 2013;3:1-150.

[24] Friedewald WT, Levy RI and Fredrickson DS. Estimation of the concentration of low-density lipoprotein cholesterol in plasma, without use of the preparative ultracentrifuge. *Clin Chem* 1972;18:499-502.

[25] RStudio Team. RStudio: Integrated Development for R. Boston, MA: RStudio, Inc., 2016.

[26] Abergel E, Tase M, Menard J, et al. Influence of obesity on the diagnostic value of electrocardiographic criteria for detecting left ventricular hypertrophy. *Am J Cardiol* 1996;77:739-744.

[27] Vlachos K, Georgopoulos S, Efremidis M, et al. An update on risk factors for drug-induced arrhythmias. *Expert Rev Clin Pharmacol* 2016;9:117-127.

[28] Agyemang C, Kunst AE, Bhopal R, et al. Brief report: diabetes prevalence in populations of South Asian Indian and African origins: a comparison of England and The Netherlands. *Epidemiology* 2011;22:563-567.

[29] Regitz-Zagrosek V and Kararigas G. Mechanistic pathways of sex differences in cardiovascular disease. *Physiol Rev* 2016;97:1-37.

[30] Mosca L, Barrett-Connor E and Kass Wenger N. Sex/gender differences in cardiovascular disease prevention: what a difference a decade makes. *Circulation* 2011;124:2145-2154.

For peer review only

For peer review only - http://bmjopen.bmj.com/site/about/guidelines.xhtml

[31] Ho JE, Paultre F and Mosca L. The gender gap in coronary heart disease mortality: is there a difference between blacks and whites? *J Womens Health* 2005;14:117-127.

[32] Shah BM, Shah S, Kandula NR, et al. Psychosocial factors associated with subclinical atherosclerosis in South Asians: the MASALA study. *J Immigr Minor Health* 2016;18:1317-1327.

[33] Rijnbeek PR, Van Herpen G, Bots ML, et al. Normal values of the electrocardiogram for ages 16-90 years. *J Electrocardiol* 2014;47:914-921.

[34] Macfarlane P, Katibi I, Hamde S, et al. Racial differences in the ECG-selected aspects. *J Electrocardiol* 2014;47:809-814.

[35] Clark E, Devine B and Macfarlane P. Age and sex dependent criteria for lower limits of QRS voltages. *2006 Computers in Cardiology*. Valencia, Spain: IEEE, 17-20 September 2006, p. 17-20.

[36] Yang T-F and Macfarlane PW. Comparison of the derived vectorcardiogram in apparently healthy whites and Chinese. *Chest* 1994;106:1014-1020.

[37] Badheka AO, Patel N, Tuliani TA, et al. Electrocardiographic abnormalities and reclassification of cardiovascular risk: insights from NHANES-III. *Am J Med* 2013;126:319-326.

[38] Auer R, Bauer DC, Marques-Vidal P, et al. Association of major and minor ECG abnormalities with coronary heart disease events. *JAMA* 2012;307:1497-1505.

[39] Jonas DE, Reddy S, Middleton JC, et al. Screening for cardiovascular disease risk with resting or exercise electrocardiography: evidence report and systematic review for the US Preventive Services Task Force. *JAMA* 2018;319:2315-2328.

For peer review only

For peer review only - http://bmjopen.bmj.com/site/about/guidelines.xhtml

FIGURE LEGEND

Figure 1. Prevalence of any major ECG abnormalities in men and women by age-groups and ethnicity.

Appendix Figure 1. Flow diagram of the study population

For peer review only

For peer review only - http://bmjopen.bmj.com/site/about/guidelines.xhtml

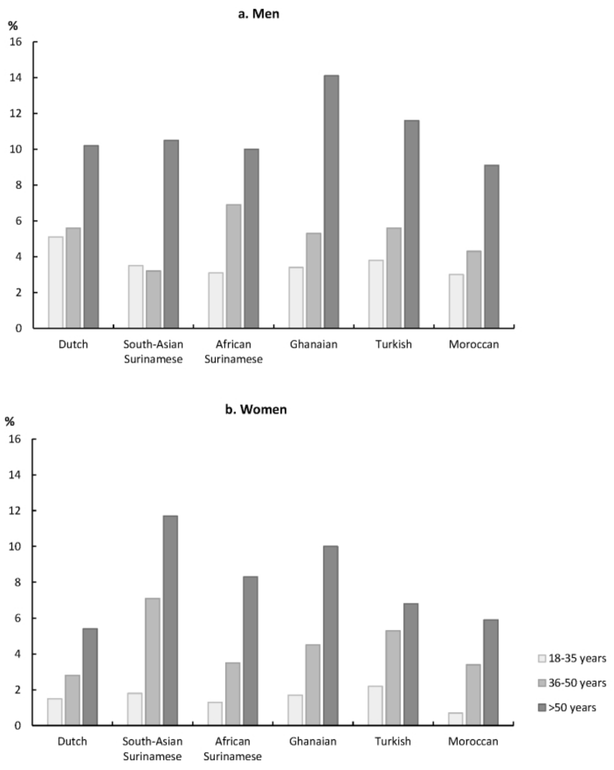

218x309mm (300 x 300 DPI)

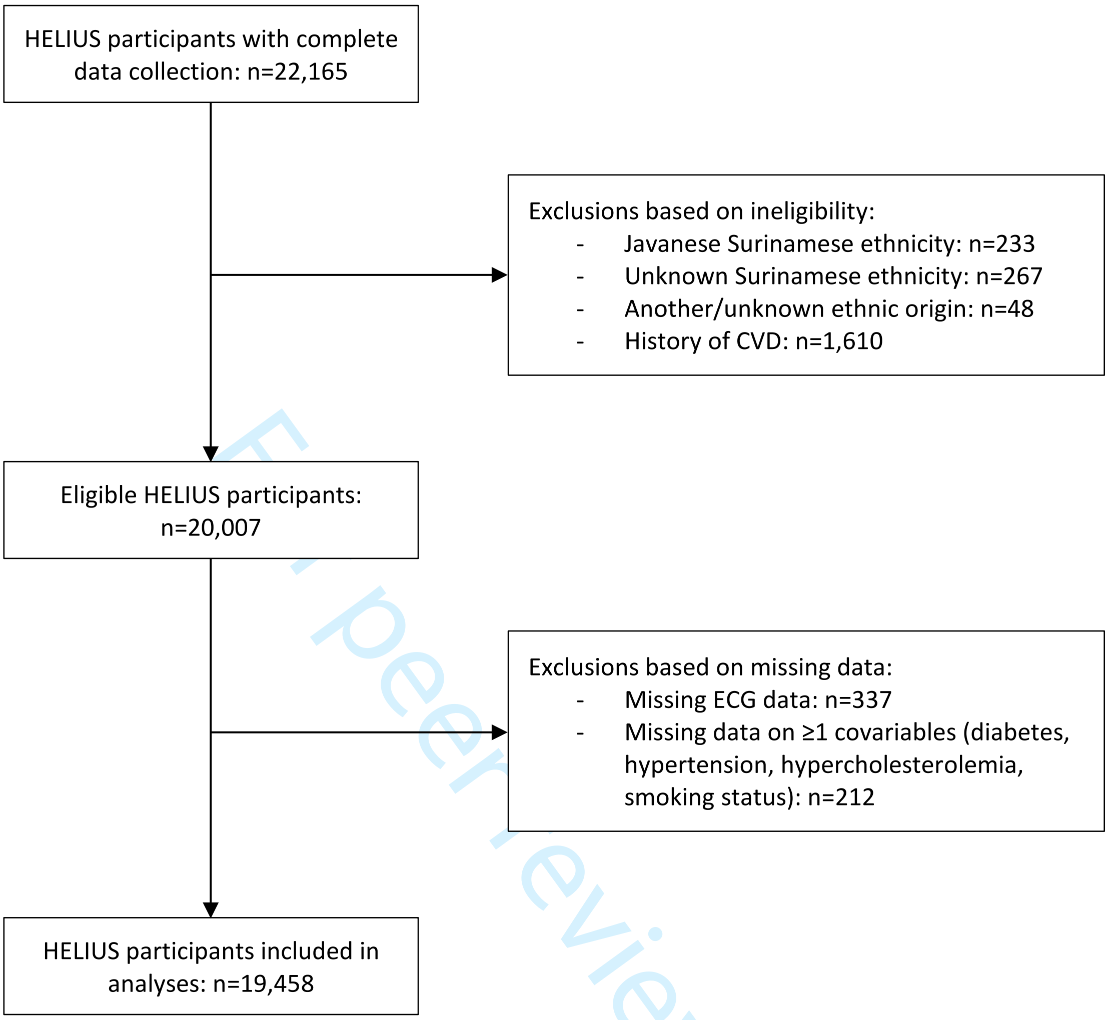

Appendix Figure 1. Flow diagram of the study population

For peer review only

Appendix Table 1. Classification of major and minor ECG abnormalities

**Major ECG abnormalities**
Atrial fibrillation or flutter
Ventricular rhythm
Sinus bradycardia + idioventricular rhythm
2nd degree atrioventricular conduction disturbances
3rd degree atrioventricular conduction disturbances
Ventricular preexcitation
Left bundle branch block (LBBB)
Right bundle branch block (RBBB)
Nonspecific ventricular conduction disturbances (QRS duration ≥120ms)
Severely prolonged QTc (Bazett) interval (men: QTc >470ms, women: QTc ≥480ms)
Severely shortened QTc (Bazett) interval (men: QTc <330ms, women: QTc <340ms)
Extreme axis deviation
Pathological Q waves
Microvoltages
T-wave abnormalities
ECG suggestive of cardiomyopathy
Miscellaneous (including ECG suggestive of Brugada syndrome)
**Minor ECG abnormalities**
Sinus tachycardia
Sinus bradycardia
Atrial rhythm
Frequent premature atrial contractions (PACs) and/or premature ventricular contractions (PVCs)
Atrial abnormalities
1st degree atrioventricular conduction disturbances
Left anterior fascicular block (LAFB)
Mild (110≤QRS<120ms) ventricular conduction disturbances, including incomplete LBBB and incomplete RBBB

For peer review only

Mildly prolonged QTc (Bazett) interval (men: QTc >450ms, women: QTc ≥460ms)

Mildly shortened QTc (Bazett) interval (men: QTc <360ms, women: QTc <370ms)

Left axis deviation

Right axis deviation

Indeterminate heart axis

Possible pathological Q waves

Left ventricular hypertrophy (LVH)

ECG, electrocardiogram.

For peer review only

Appendix Table 2. Baseline characteristics of 19,548 participants with ECG measurements, by sex and ethnicity

	Dutch		South-Asian Surinamese		African Surinamese	
	**Men (n=1,873)**	**Women (n=2,293)**	**Men (n=1,125)**	**Women (n=1,464)**	**Men (n=1,411)**	**Women (n=2,266)**
Age (years)	45.8 (13.6)	44.8 (14.0)	42.6 (13.2)	45.0 (13.1)	47.2 (12.9)	47.1 (12.3)
Family history of CVD (missing: n=217*)	418 (22.4)	536 (23.5)	399 (35.8)	583 (40.5)	231 (16.5)	473 (21.2)
Smoker						
Current	477 (25.5)	532 (23.2)	432 (38.4)	277 (18.9)	597 (42.3)	548 (24.2)
Past	722 (38.5)	842 (36.7)	177 (15.7)	159 (10.9)	291 (20.6)	400 (17.7)
Never	674 (36.0)	919 (40.1)	516 (45.9)	1,028 (70.2)	523 (37.1)	1,318 (58.2)
Pack-years of smoking (missing: n=191*)	3.4 (10.6)	2.9 (9.7)	8.9 (26.9)	1.7 (5.9)	8.3 (20.3)	2.5 (9.8)
Achieving physical activity norm (missing: n=27*)	1,365 (72.9)	1,792 (78.2)	645 (57.5)	729 (49.9)	983 (69.8)	1,270 (56.1)
Alcohol consumption (missing: n=115*)						
None or low	721 (38.6)	774 (33.9)	880 (78.6)	1,261 (86.7)	1,056 (75.6)	1,883 (83.7)
Moderate	884 (47.3)	925 (40.5)	151 (13.5)	149 (10.2)	260 (18.6)	278 (12.4)

For peer review only

High	264 (14.1)	587 (25.7)	89 (7.9)	44 (3.0)	80 (5.7)	89 (4.0)
BMI (kg/m^2; missing: n=15*)	25.0 (3.7)	24.2 (4.3)	25.7 (4.1)	26.4 (5.2)	26.2 (4.3)	28.6 (5.9)
CKD risk (missing: n=63*)						
Low	1,804 (96.8)	2,216 (96.9)	1,046 (93.2)	1,365 (93.5)	1,344 (95.5)	2,139 (94.6)
Moderate	49 (2.6)	64 (2.8)	62 (5.5)	78 (5.3)	53 (3.8)	100 (4.4)
High	10 (0.5)	7 (0.3)	14 (1.2)	17 (1.2)	11 (0.8)	21 (0.9)
Hypertension	618 (33.0)	471 (20.5)	429 (38.1)	527 (36.0)	660 (46.8)	1,072 (47.3)
Hypercholesterolemia	1,314 (70.2)	1,374 (59.9)	934 (83.0)	1,120 (76.5)	889 (63.0)	1,447 (63.9)
Diabetes	76 (4.1)	40 (1.7)	190 (16.9)	209 (14.3)	149 (10.6)	237 (10.5)
Use of psychotropic medication (missing: n=4*)	88 (4.7)	190 (8.3)	57 (5.1)	100 (6.8)	53 (3.8)	105 (4.6)
	**Ghanaian**		**Turkish**		**Moroccan**	
	**Men (n=822)**	**Women (n=1,321)**	**Men (n=1,451)**	**Women (n=1,769)**	**Men (n=1,407)**	**Women (n=2,256)**
Age (years)	46.3 (11.6)	43.0 (10.7)	39.8 (11.9)	39.1 (12.0)	41.6 (12.7)	39.1 (12.8)
Family history of CVD (missing: n=217*)	36 (4.4)	53 (4.1)	382 (26.7)	615 (35.2)	171 (12.3)	351 (15.8)
Smoker						
Current	60 (7.3)	32 (2.4)	602 (41.5)	521 (29.5)	371 (26.4)	122 (5.4)

Past	102 (12.4)	66 (5.0)	354 (24.4)	207 (11.7)	375 (26.7)	79 (3.5)
Never	660 (80.3)	1,223 (92.6)	495 (34.1)	1,041 (58.8)	661 (47.0)	2,055 (91.1)
Pack-years of smoking (missing: n=191*)	0.6 (4.2)	0.2 (2.3)	6.9 (14.8)	2.9 (7.2)	3.8 (9.9)	0.3 (1.9)
Achieving physical activity norm (missing: n=27*)	516 (62.8)	626 (47.4)	720 (49.8)	618 (35.0)	791 (56.4)	928 (41.2)
Alcohol consumption (missing: n=115*)						
None or low	711 (87.6)	1,184 (90.5)	1,291 (89.8)	1,679 (95.3)	1,322 (94.4)	2,204 (98.0)
Moderate	98 (12.1)	103 (7.9)	87 (6.1)	62 (3.5)	46 (3.3)	32 (1.4)
High	3 (0.4)	22 (1.7)	59 (4.1)	20 (1.1)	33 (2.4)	12 (0.5)
BMI (kg/m^2; missing: n=15*)	26.6 (3.6)	29.5 (5.3)	27.7 (4.4)	28.9 (6.5)	26.7 (4.0)	28.0 (5.7)
CKD risk (missing: n=63*)						
Low	770 (94.2)	1,213 (92.2)	1,379 (95.4)	1,645 (93.3)	1,341 (95.7)	2,111 (93.7)
Moderate	37 (4.5)	87 (6.6)	57 (3.9)	106 (6.0)	46 (3.3)	120 (5.3)
High	10 (1.2)	16 (1.2)	9 (0.6)	12 (0.7)	14 (1.0)	22 (1.0)
Hypertension	494 (60.1)	673 (50.9)	427 (29.4)	407 (23.0)	398 (28.3)	444 (19.7)
Hypercholesterolemia	551 (67.0)	792 (60.0)	1,120 (77.2)	1,130 (63.9)	944 (67.1)	1,284 (56.9)

For peer review only

Diabetes	115 (14.0)	110 (8.3)	138 (9.5)	134 (7.6)	161 (11.4)	222 (9.8)
Use of psychotropic medication (missing: n=4*)	24 (2.9)	41 (3.1)	88 (6.1)	139 (7.9)	87 (6.2)	104 (4.6)

Data are presented as means (standard deviations) or frequencies (percentages).

BMI, body mass index; CKD, chronic kidney disease; CVD, cardiovascular disease.

* From total study population (n=19,458).

For peer review only

Appendix Table 3. Number of cases and age-adjusted prevalence of any minor ECG abnormality by sex in the total population and by ethnic group, and the odds of minor ECG abnormalities in women compared to men, overall and with an interaction term for sex and ethnicity

	**Men**	**Women**	**Model 1**				**Model 2**			
	**(n of cases, %[a])**	**(n of cases, %[a])**	**OR (95% CI)**	***p*-value**	**Ratio of ORs (95% CI)***	***p*-value**	**OR (95% CI)**	***p*-value**	**Ratio of ORs (95% CI)***	***p*-value**
Overall	3,227 (39.8)	2,684 (23.8)	0.45 (0.42-0.48)[b]	*<0.001*	NA	NA	0.46 (0.43-0.49)[b]	*<0.001*	NA	NA
Dutch	829 (44.3)	636 (27.6)	0.49 (0.43-0.55)	*<0.001*	Reference	NA	0.50 (0.44-0.57)	*<0.001*	Reference	NA
SA Surinamese	316 (28.5)	269 (17.9)	0.56 (0.46-0.67)	*<0.001*	1.15 (0.92-1.44)	0.22	0.57 (0.47-0.68)	*<0.001*	1.13 (0.90-1.42)	0.28
African Surinamese	647 (45.5)	638 (28.4)	0.46 (0.40-0.53)	*<0.001*	0.95 (0.78-1.15)	0.59	0.46 (0.40-0.53)	*<0.001*	0.93 (0.76-1.12)	0.43
Ghanaian	455 (55.7)	452 (35.6)	0.43 (0.36-0.52)	*<0.001*	0.89 (0.72-1.11)	0.31	0.44 (0.36-0.52)	*<0.001*	0.87 (0.70-1.09)	0.22
Turkish	468 (32.4)	283 (16.2)	0.40 (0.34-0.48)	*<0.001*	0.83 (0.67-1.02)	0.08	0.40 (0.34-0.47)	*<0.001*	0.80 (0.64-0.99)	*0.04*
Moroccan	512 (36.5)	406 (18.3)	0.39 (0.34-0.46)	*<0.001*	0.81 (0.66-0.99)	*0.04*	0.39 (0.34-0.46)	*<0.001*	0.78 (0.64-0.96)	*0.02*

CI, confidence interval; NA, not applicable; OR, odds ratio; SA, South-Asian.

Significant *p*-values ($p<0.05$) are printed in italic.

Model 1: adjusted for age.

Model 2: adjusted for age, hypertension, hypercholesterolemia, diabetes, and smoking status.

[a] Age-adjusted prevalence.

For peer review only

[b] Additionally adjusted for ethnicity.

* Measure of effect modification on multiplicative scale (statistical interaction term).

For peer review only

Appendix Table 4a. Age-adjusted prevalence of a selection of common ECG abnormalities (for majors: prevalence ≥1% in total study population; for minors: top 5 of abnormalities with highest prevalence in total study population), shown by major and minor ECG abnormality categories, by sex and ethnicity, ordered by highest to lowest overall prevalence

	**Major ECG abnormalities**		**Minor ECG abnormalities**				
	**T-wave abnormalities**	**ECG suggestive of cardiomyopathy**	**LVH**	**Sinus bradycardia**	**1st degree atrioventricular conduction disturbances**	**Mild ventricular conduction disturbances[a]**	**Mildly prolonged QTc (Bazett) interval[b]**
All							
Men	133 (1.6)	90 (1.1)	1,088 (13.5)	723 (9.0)	532 (6.5)	609 (7.5)	173 (2.1)
Women	137 (1.2)	124 (1.1)	986 (8.8)	473 (4.2)	344 (3.1)	133 (1.2)	393 (3.5)
Dutch							
Men	7 (0.4)	10 (0.5)	181 (9.8)	269 (14.3)	121 (6.3)	254 (13.6)	57 (3.0)
Women	12 (0.5)	15 (0.6)	114 (5.0)	218 (9.4)	72 (3.1)	55 (2.4)	69 (3.1)
South-Asian Surinamese							
Men	17 (1.9)	13 (1.2)	79 (7.2)	78 (6.9)	36 (3.4)	42 (3.9)	23 (2.1)
Women	23 (1.5)	28 (1.9)	100 (6.5)	40 (2.8)	22 (1.5)	3 (0.2)	56 (3.6)

For peer review only

	Major ECG abnormalities		Minor ECG abnormalities				
	T-wave abnormalities	ECG suggestive of cardiomyopathy	LVH	Sinus bradycardia	1st degree atrioventricular conduction disturbances	Mild ventricular conduction disturbances[a]	Mildly prolonged QTc (Bazett) interval[b]
African Surinamese							
Men	50 (3.5)	38 (2.7)	288 (20.3)	124 (8.8)	137 (9.5)	62 (4.3)	33 (2.3)
Women	47 (2.1)	41 (1.9)	340 (15.1)	63 (2.8)	102 (4.5)	16 (0.7)	94 (4.2)
Ghanaian							
Men	40 (4.3)	11 (1.2)	293 (35.5)	41 (5.4)	103 (12.9)	22 (2.5)	9 (1.1)
Women	32 (2.7)	25 (2.1)	266 (21.3)	42 (3.4)	79 (6.1)	7 (0.6)	29 (2.5)
Turkish							
Men	13 (0.9)	10 (0.7)	87 (6.0)	89 (6.2)	55 (3.8)	118 (8.2)	37 (2.5)
Women	11 (0.7)	7 (0.4)	58 (3.3)	36 (2.0)	23 (1.3)	23 (1.4)	69 (3.9)
Moroccan							
Men	6 (0.4)	8 (0.5)	160 (11.5)	122 (9.1)	80 (5.3)	111 (7.8)	14 (0.9)
Women	12 (0.6)	8 (0.4)	108 (4.9)	74 (3.2)	46 (2.1)	29 (1.3)	76 (3.6)

Data are reported in n (%). ECG, electrocardiogram.

[a] 110≤QRS<120ms; [b] men: QTc >450ms, women: QTc ≥460ms.

For peer review only - http://bmjopen.bmj.com/site/about/guidelines.xhtml

Appendix Table 4b. Age-adjusted prevalence of less common ECG abnormalities (for majors: prevalence <1% in total study population; for minors: abnormalities not in top 5 with highest prevalence in total study population), shown by major and minor ECG abnormality categories, by sex, ordered by highest to lowest overall prevalence

	**Men**	**Women**
**Major ECG abnormalities**		
Microvoltages	38 (0.5)	138 (1.2)
Right bundle branch block (RBBB)	117 (1.4)	38 (0.3)
Severely prolonged QTc (Bazett) interval[a]	59 (0.7)	91 (0.8)
Nonspecific ventricular conduction disturbances[b]	87 (1.1)	10 (0.1)
Pathological Q waves	50 (0.6)	32 (0.3)
Left bundle branch block (LBBB)	20 (0.2)	36 (0.3)
Ventricular preexcitation	27 (0.3)	16 (0.1)
Extreme axis deviation	15 (0.2)	7 (0.06)
Atrial fibrillation or flutter	8 (0.1)	5 (0.05)
Miscellaneous[c]	7 (0.08)	2 (0.02)
Severely shortened QTc (Bazett) interval[d]	3 (0.04)	2 (0.02)
Ventricular rhythm	2 (0.02)	1 (0.01)
2nd degree atrioventricular conduction disturbances	3 (0.04)	0
Sinus bradycardia + idioventricular rhythm	2 (0.02)	0
3rd degree atrioventricular conduction disturbances	0	0
**Minor ECG abnormalities**		
Left axis deviation	371 (4.5)	207 (1.9)
Right axis deviation	222 (2.8)	120 (1.1)
Possible pathological Q waves	172 (2.1)	111 (1.0)
Frequent PACs and/or PVCs	99 (1.2)	118 (1.1)
Atrial rhythm	79 (1.0)	130 (1.1)
Mildly shortened QTc (Bazett) interval[e]	1.7 (1.4)	100 (0.9)

For peer review only

	Men	Women
Left anterior fascicular block (LAFB)	61 (0.7)	32 (0.3)
Sinus tachycardia	23 (0.3)	37 (0.3)
Atrial abnormalities	32 (0.4)	27 (0.2)
Indeterminate heart axis	9 (0.1)	4 (0.03)

Data are reported in n (%).

ECG, electrocardiogram; PAC, premature atrial contraction; PVC, premature ventricular contraction.

[a] men: QTc >470ms, women: QTc ≥480ms;

[b] QRS duration ≥120ms.

[c] Including ECG suggestive of Brugada syndrome.

[d] men: QTc <330ms, women: QTc <340ms.

[e] men: QTc <360ms, women: QTc <370ms

For peer review only

STROBE 2007 (v4) Statement—Checklist of items that should be included in reports of *cross-sectional studies*

Section/Topic	Item #	Recommendation	Reported on page #
**Title and abstract**	1	(*a*) Indicate the study's design with a commonly used term in the title or the abstract	1
		(*b*) Provide in the abstract an informative and balanced summary of what was done and what was found	2, 3
**Introduction**			
Background/rationale	2	Explain the scientific background and rationale for the investigation being reported	6
Objectives	3	State specific objectives, including any prespecified hypotheses	7
**Methods**			
Study design	4	Present key elements of study design early in the paper	8
Setting	5	Describe the setting, locations, and relevant dates, including periods of recruitment, exposure, follow-up, and data collection	8
Participants	6	(*a*) Give the eligibility criteria, and the sources and methods of selection of participants	8, 10
Variables	7	Clearly define all outcomes, exposures, predictors, potential confounders, and effect modifiers. Give diagnostic criteria, if applicable	8, 9
Data sources/ measurement	8*	For each variable of interest, give sources of data and details of methods of assessment (measurement). Describe comparability of assessment methods if there is more than one group	8, 9
Bias	9	Describe any efforts to address potential sources of bias	8, 10, 11
Study size	10	Explain how the study size was arrived at	10
Quantitative variables	11	Explain how quantitative variables were handled in the analyses. If applicable, describe which groupings were chosen and why	8-11
Statistical methods	12	(*a*) Describe all statistical methods, including those used to control for confounding	10, 11
		(*b*) Describe any methods used to examine subgroups and interactions	10, 11
		(*c*) Explain how missing data were addressed	10
		(*d*) If applicable, describe analytical methods taking account of sampling strategy	NA
		(*e*) Describe any sensitivity analyses	11
**Results**			

For peer review only

Participants	13*	(a) Report numbers of individuals at each stage of study—eg numbers potentially eligible, examined for eligibility, confirmed eligible, included in the study, completing follow-up, and analysed	10
		(b) Give reasons for non-participation at each stage	NA
		(c) Consider use of a flow diagram	
Descriptive data	14*	(a) Give characteristics of study participants (eg demographic, clinical, social) and information on exposures and potential confounders	13
		(b) Indicate number of participants with missing data for each variable of interest	13, 14
Outcome data	15*	Report numbers of outcome events or summary measures	14, 16-19
Main results	16	(*a*) Give unadjusted estimates and, if applicable, confounder-adjusted estimates and their precision (eg, 95% confidence interval). Make clear which confounders were adjusted for and why they were included	Table 2, Table 3, and Appendix Table 3
		(*b*) Report category boundaries when continuous variables were categorized	17, 18
		(*c*) If relevant, consider translating estimates of relative risk into absolute risk for a meaningful time period	NA
Other analyses	17	Report other analyses done—eg analyses of subgroups and interactions, and sensitivity analyses	15-19
**Discussion**			
Key results	18	Summarise key results with reference to study objectives	20
Limitations	19	Discuss limitations of the study, taking into account sources of potential bias or imprecision. Discuss both direction and magnitude of any potential bias	20, 21
Interpretation	20	Give a cautious overall interpretation of results considering objectives, limitations, multiplicity of analyses, results from similar studies, and other relevant evidence	21-24
Generalisability	21	Discuss the generalisability (external validity) of the study results	20
**Other information**			
Funding	22	Give the source of funding and the role of the funders for the present study and, if applicable, for the original study on which the present article is based	25

*Give information separately for cases and controls in case-control studies and, if applicable, for exposed and unexposed groups in cohort and cross-sectional studies.

Note: An Explanation and Elaboration article discusses each checklist item and gives methodological background and published examples of transparent reporting. The STROBE checklist is best used in conjunction with this article (freely available on the Web sites of PLoS Medicine at http://www.plosmedicine.org/, Annals of Internal Medicine at http://www.annals.org/, and Epidemiology at http://www.epidem.com/). Information on the STROBE Initiative is available at www.strobe-statement.org.

For peer review only
